# Aberrant MEG multi-frequency phase temporal synchronization predicts conversion from mild cognitive impairment-to-Alzheimer's disease

**DOI:** 10.1016/j.nicl.2019.101972

**Published:** 2019-08-08

**Authors:** Sandra Pusil, Stavros I. Dimitriadis, María Eugenia López, Ernesto Pereda, Fernando Maestú

**Affiliations:** a Laboratory of Neuropsychology, University of the Balearic Islands, Spain; b Laboratory of Cognitive and Computational Neuroscience, Center for Biomedical Technology, Universidad Complutense and Universidad Politécnica de Madrid, Madrid, Spain; c Cardiff University Brain Research Imaging Centre, School of Psychology, Cardiff University, Cardiff, United Kingdom; d Neuroinformatics Group, Cardiff University Brain Research Imaging Centre, School of Psychology, Cardiff University, Cardiff, United Kingdom; e Division of Psychological Medicine and Clinical Neurosciences, School of Medicine, Cardiff University, Cardiff, United Kingdom; f School of Psychology, Cardiff University, Cardiff, United Kingdom; g Neuroscience and Mental Health Research Institute, School of Medicine, Cardiff University, Cardiff, United Kingdom; h MRC Centre for Neuropsychiatric Genetics and Genomics, School of Medicine, Cardiff University, Cardiff, United Kingdom; i Department of Experimental Psychology, Universidad Complutense de Madrid, Madrid, Spain; j Networking Research Center on Bioengineering, Biomaterials and Nanomedicine (CIBER-BBN), Madrid, Spain; k Electrical Engineering and Bioengineering Lab, Department of Industrial Engineering, IUNE Universidad de La Laguna, Tenerife, Spain

**Keywords:** Mild cognitive impairment, Conversion, Magnetoencephalography, Source reconstruction, Dynamic functional connectivity analysis, Connectomic biomarker

## Abstract

Many neuroimaging studies focus on a frequency-specific or a multi-frequency network analysis showing that functional brain networks are disrupted in patients with Alzheimer's disease (AD). Although those studies enriched our knowledge of the impact of AD in brain's functionality, our goal is to test the effectiveness of combining neuroimaging with network neuroscience to predict with high accuracy subjects with mild cognitive impairment (MCI) that will convert to AD.

In this study, eyes-closed resting-state magnetoencephalography (MEG) recordings from 27 stable MCI (sMCI) and 27 progressive MCI (pMCI) from two scan sessions (baseline and follow-up after approximately 3 years) were projected via beamforming onto an atlas-based set of regions of interest (ROIs). Dynamic functional connectivity networks were constructed independently for the five classical frequency bands while a multivariate phase-based coupling metric was adopted. Thus, computing the distance between the fluctuation of functional strength of every pair of ROIs between the two conditions with dynamic time wrapping (DTW), a large set of features was extracted. A machine learning algorithm revealed 30 DTW-based features in the five frequency bands that can distinguish the sMCI from pMCI with absolute accuracy (100%). Further analysis of the selected links revealed that most of the connected ROIs were part of the default mode network (DMN), the cingulo-opercular (CO), the fronto-parietal and the sensorimotor network.

Overall, our dynamic network multi-frequency analysis approach provides an effective framework of constructing a sensitive MEG-based connectome biomarker for the prediction of conversion from MCI to Alzheimer's disease.

## Highlights

• 49 features in the five frequency bands discriminated the two groups with 100% accuracy.
• The analysis revealed most of the links were part of important brain networks as the DMN.
• The alteration of these links, usually affected in AD, is related to synaptic loss.
• Our multi-frequency approach provided a MEG connectome biomarker to predict the conversion to AD.

## 1 Introduction

Alzheimer's disease (AD) is a neurodegenerative disease, currently considered the most common type of dementia corresponding to 60–70% of the cases in the world population (Wimo et al., 1997). This disease is clinically defined by a progressive loss of episodic memory and other cognitive and functional abilities, such as executive functions (Guarino et al., 2019). Histologically, AD is characterized by the presence of amyloid plaques, neurofibrillary tangles and brain atrophy (Braak and Braak, 1991).

The neurodegenerative cascade in AD begins decades before the clinical and neuroimaging manifestations of the disease are evident (Jr et al., 2010). Therefore, establish an early diagnosis is of great significance to initiate pharmacological or cognitive treatments that may slow down the progression of the disorder. One of the most studied phases in the prognosis of AD is Mild Cognitive Impairment (MCI), since it entails a higher risk of developing Alzheimer-type dementia (Shah et al., 2000; Farias et al., 2005, Petersen et al., 2001). In fact, several longitudinal studies have found that the conversion rate from MCI to AD is 10–15%/year (Mitchell and Shiri-Feshki, 2009). However, the progression of this transitional stage between normal aging and dementia has usually been quite variable due to its heterogeneous nature. The inclusion of more precise clinical criteria and biomarker's characteristics is very important to get an increasingly accurate diagnosis and prognosis.

In recent years, different neuroimaging modalities have studied AD progression (Cui et al., 2011; Teipel et al., 2015; Zhang et al., 2014). In particular, the magnetoencephalography (MEG), which provides an effective and non-invasive way to capture human brain's functional connectivity (FC) patterns (Brookes et al., 2011). In this way, the multivariate phase coupling estimation (PCE) provides a new approach to reveal the functional coupling based on multivariate phase statistics between nodes in a large network (Cadieu and Koepsell, 2010). MEG FC networks are thus a promising approach to characterize brain organization under both healthy and pathological conditions.

Recently, graph analysis has been used to classify subjects from different populations (Tijms et al., 2014). A common problem of this approach is that most of the studies that used network properties established an arbitrary threshold from the original weighted network as the sorting variable. In order to solve these limitations, new techniques such as the Orthogonal Minimal Spanning Tree (Dimitriadis et al., 2017a; Dimitriadis et al., 2017b; Dimitriadis and Salis, 2017b) are currently being implemented in network neuroscience. In this line, we have recently published an extensive work of the different choices during the preprocessing steps of MEG resting-state activity tailored to the design of a reliable connectomic biomarker for MCI (Dimitriadis et al., 2018a). Complementary, we have also explored the reliability of both static and dynamic network metrics of source-reconstructed neuromagnetic activity at resting-state, obtaining that static network metrics are less reliable than dynamic's (Dimitriadis et al., 2018b). In our previous analysis, we adopted two commonly used bivariate connectivity estimators, the imaginary part of phase locking value (iPLV: Bruña et al., 2018; Dimitriadis et al., 2018a) and the orthogonalized correlation of the envelope (CorrEnv; Dimitriadis et al., 2018a).

Many prospective studies have focused on the progression from MCI to AD (Aguilar et al., 2013; Cui et al., 2018, 2011; Rasero et al., 2017). For instance, in a recent MEG study of our group found that the increase in phase synchronization between the right anterior cingulate and temporo-occipital areas together with the immediate recall score in MCI patients predicted the conversion to AD with an accuracy of 89.9% (López et al., 2014a). Nevertheless, there is a scarcity of longitudinal investigations that have used repeated MEG measurements of these subjects over time.

In the present study, we used a multivariate connectivity estimator focused on the multivariate phase of the multi-source activity called phase coupling estimation (PCE) (Cadieu and Koepsell, 2010). And moved one further step by adopting well-known graph signal processing operators called Laplacian transformations (Chung, 2005). A previous study analyzed the structural connectivity matrices from healthy controls, early/late MCI and AD (Daianu et al., 2014). These authors explored the network's algebraic connectivity via graph Laplacian spectrum and the Fiedler value, which is the second smallest eigenvalue of the Laplacian matrix. By using this approach, they found reduced structural network robustness in AD. Focusing on a Laplacian matrix derived from the functional brain network, they defined a biomarker based on the Laplacian graph operator (Van Mieghem, 2011).

Thus, in the present work, we studied a sample of MCIs that were followed-up during an approximate 3-year period. The first MEG scan was done when all participants were MCI (first condition or session); the second one (second condition or session) when some of them had progressed to AD (*progressive* MCI, pMCI) while others had remained as MCI in the same period (*stable* MCI, sMCI). We first used a multivariate phase coupling estimator applied to frequency dependent source-reconstructed brain activity to quantify dynamic whole-brain functional connectivity brain graphs in both conditions. Dynamic Time Wrapping (DTE) was adopted as a distance metric to estimate the (dis)similarity of fluctuations of functional coupling strength between the two conditions and for every pair of ROIs across frequencies. A classification framework extracted the most informative multi-frequency topological features supporting an appropriate classifier that succeeded to predict subjects that converted from MCI to AD.

Section 1 is devoted to describing the dataset, the demographics, and the analytic pathway. Section 2 demonstrates the novel results dedicated to the current protocol with follow-up. Finally, Section 3 is devoted to the discussion of current findings linked to the current literature and proposing complementary research directions.

## 2 Materials and methods

### 2.1 Subjects

MEG recordings were obtained from 54 MCI patients recruited from the Hospital Universitario San Carlos (Madrid, Spain). All of them were right-handed (Oldfield, 1971). In Table 1 we introduced their demographic data.

**Table 1:** Mean ± SD values of the demographic characteristics of the sMCI and pMCI patients at baseline. MMSE: Mini-Mental State Examination; Apolipoprotein E (APOE) carrier: There is at least 1 ε4 allele; LHV: Left hippocampal volume; RHV: Right hippocampal volume.

	sMCI (n = 27)	pMCI (n = 27)	F-value	*p*-value
Age (years)	71,23 ± 3,98	74,81 ± 3,98	26,137	0,009*
Gender (females)	15	18	Fisher test	0,577
APOE 4 carrier	12	13	Fisher test	0,782
Education (years)	8,88 ± 4,49	8,6 ± 4,49	0,0064	0,937
MMSE score (first MEG)	27,34 ± 3,39	25,95 ± 3,39	32,289	0,079
MMSE (second MEG)	26,19 ± 4,13	23,65 ± 4,13	29,490	0,092
LHV	0,0024 ± 0,0003	0,0020 ± 0,0003	97,773	0,003*
RHV	0,0025 ± 0,0003	0,0022 ± 0,0003	55,714	0,023*

*p-values* for between-groups differences were introduced, and *p < .05. Age differences were assessed with a Mann-Whitney Test. An ANCOVA test, with age as a co-variable, was used for continuous variables and Fisher's exact test for gender and APOE differences.

MCI diagnosis was made according to the National Institute on Aging-Alzheimer Association (NIA-AA) clinical criteria (Albert et al., 2011). Besides meeting the clinical criteria, MCI participants had signs of neuronal injury (hippocampal volume measured by magnetic resonance imaging (MRI). Thus, they might be considered as “MCI due to AD” with an intermediate likelihood (Albert et al., 2011). Besides, they were cognitively and clinically followed-up for approximately three years (every six months) and were then split into two groups according to their clinical outcome: 1) the “progressive” MCI group (pMCI; *n* = 27) was composed of those subjects that met the criteria for probable AD (McKhann et al., 2011) and 2) the “stable” MCI group (sMCI; *n* = 27) was comprised of those participants that still fulfilled the diagnosis criteria of MCI at the end of follow-up.

None of the participants had a history of psychiatric or neurological disorders (other than MCI or AD). General inclusion criteria were: age between 65 and 80, a modified Hachinski score ≤ 4, a short-form Geriatric Depression Scale score ≤ 5, and T1 magnetic resonance imaging (MRI) within 12 months and 2 weeks before the two MEG recordings without indication of infection, infarction, or focal lesions (rated by two independent experienced radiologists; (Bai et al., 2012). Patients were off those medications that could affect MEG activity, such as cholinesterase inhibitors, 48 h before recordings.

The study was approved by the Hospital Universitario San Carlos Ethics Committee (Madrid), and all participants signed a written informed consent prior to participation.

### 2.2 MRI and medial temporal lobe volumes

3D T1 weighted anatomical brain magnetic resonance imaging (MRI) scans were collected with a General Electric 1.5 T MRI scanner, using a high-resolution antenna and a homogenization PURE filter (Fast Spoiled Gradient Echo (FSPGR) sequence with parameters: TR/TE/TI = 11.2/4.2/ 450 ms; flip angle 12°; 1 mm slice thickness, a 256 × 256 matrix and FOV 25 cm).

We employed Freesurfer software (version 5.1.0.21) to obtain the medial temporal lobe volumes, which were normalized to the overall intracranial volume to account for differences in head volume over subjects.

### 2.3 MEG recordings

**Fig. 1:**
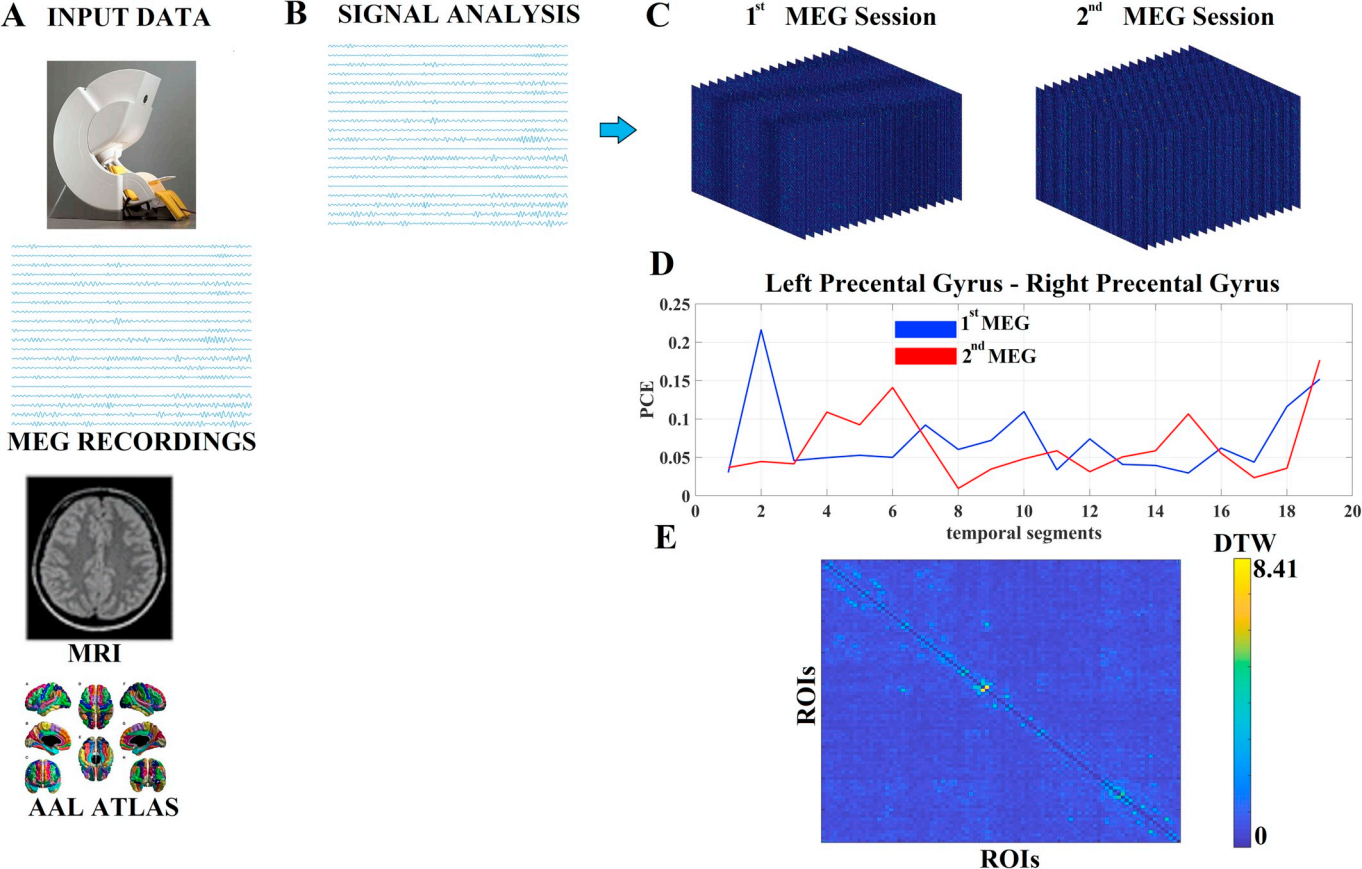
Outline of the proposed analytic scheme. A. From MEG space to source-reconstructed virtual anatomical space. B. Virtual representative frequency-dependent time series per ROI. C. dFCG of the first and second MEG across the 19 temporal segments of 4 s. D. DTW temporal distance metric between two time series representing the fluctuation of functional strength of left and right precentral gyrus in the first and second MEG sessions. An example from the δ frequency of the 1st healthy control subjects. E. DTW-based matrix tabulates the 4005 DTW distances between every possible pair of ROIs by comparing the functional strength time series in the first and second MEG sessions. An example from the δ frequency of the 1st healthy control subjects.

MEG recordings were acquired with a 306-channel Vectorview system (Elekta-Neuromag) at the Center for Biomedical Technology (Madrid, Spain). Data were collected at a sampling frequency of 1000 Hz and band-pass filtered online between 0.1 and 330 Hz (Fig. 1A).

MEG signals were recorded at the same time of the day in two different moments: 1) at baseline (first MEG), and 2) 24 ± 6 months (second MEG). Patients were in an awake, resting state with their eyes closed. For each subject, 5 min task-free data were recorded. Maxfilter software (v 2.2, correlation threshold = 0.9, time window = 10 s) was used to remove external noise of the raw MEG data with the temporal extension of the signal space separation method with movement compensation (Taulu and Simola, 2006). MEG data were automatically scanned for ocular, muscle, and jump artifacts using the Fieldtrip software (Oostenveld et al., 2011). Subsequently, artifacts were visually confirmed by a MEG expert. The remaining artifact-free data were segmented in 4 s segments (epochs). An independent component analysis-based procedure was used to remove the heart magnetic field artifact. Previously to source data calculation, MEG time series were filtered into delta δ (2–4 Hz), theta θ (4–8 Hz), alpha α (8–12 Hz), beta β (12–30 Hz), and gamma γ (30–55 Hz) frequency bands with a 1500 order finite impulse response filter with Hamming window and a two-pass filtering procedure.

### 2.4 Source reconstruction and connectivity analysis

A regular grid with 10 mm spacing was created in the template MNI. This set of nodes was transformed to each participant's space using a non-linear normalization between the native T1 image (whose coordinate system was previously converted to match the MEG coordinate system) and a standard T1 in MNI space. The forward model was solved with a single-shell method (Nolte, 2003) with a unique boundary defined by the inner skull (the combination of white matter, gray matter, and cerebrospinal fluid) taken from the individual T1. We carried out the source reconstruction independently for each subject and frequency band, using a linearly constrained minimum variance (LCMV) beamformer (Van Veen et al., 1997). Beamforming filters were estimated with normalized leadfields, regularized covariance matrices averaged over trials, and a 1% regularization factor. These neural MEG sources were anatomically parcellated by dividing the cortex into 90 regions of interest (ROIs) according to the AAL atlas (Tzourio-Mazoyer et al., 2002). We selected a centroid (CENT) and principal component analysis (PCA) the representative time series for each brain area (Fig. 1B; Dimitriadis et al., 2018a). Finally, the functional connectivity (FC) was assessed using the multivariate phase coupling estimation (PCE), that evaluates the distribution of phase differences extracted between the whole set of 90 ROIs (Cadieu and Koepsell, 2010).

We constructed a dynamic functional connectivity graph (dFCG) separately for every subject, and for the first and second MEG and frequency bands by analyzing the first 19 epochs of 4 s from the resting-state (Dimitriadis et al., 2018a, Dimitriadis et al., 2018b; Dimitriadis et al., 2017a; Dimitriadis et al., 2017b; Dimitriadis and Salis, 2017). The outcome of this procedure is a full-weighted dFCG of size 19 × 90 × 90 (Fig. 1C). The weights of the produced FCG were normalized within the range [0,1] with the maximum observed functional coupling strength. At a second level, we estimated the temporal distance between the two time series expressing the fluctuation of functional strength of a pair of ROIs in the first and second (follow-up) MEG sessions. As a proper temporal distance metric, we adopted a dynamic time wrapping (DTW) employing Symmetric Kullback-Leibler metric.

DTW has been mainly used in biomedical research to classify signals into different categories by comparing the signals with standard templates (Forestier et al., 2012). Another study introduced DTW as a task-based functional similarity between MEG sensor time series (Karamzadeh et al., 2013). Here, we employed DTW to quantify the similarity of the fluctuation of dynamic coupling strength of a pair of brain areas between 1st and 2nd MEG session.

In Fig. 1D, we demonstrated the PCE functional strength between left and right precentral gyrus across the 19 temporal segments in the first and second MEG session. Finally, we computed the DTW temporal functional strength distance for every pair of ROIs (4005 possible pairs of the 90 ROIs) between the first and second MEG leading to a matrix 90 × 90 per frequency band and subject (Fig. 1E).

Fig. 1 illustrates the preprocessing steps for analyzing neuromagnetic recordings.

### 2.5 Feature selection and classification problem

**Fig. 2:**
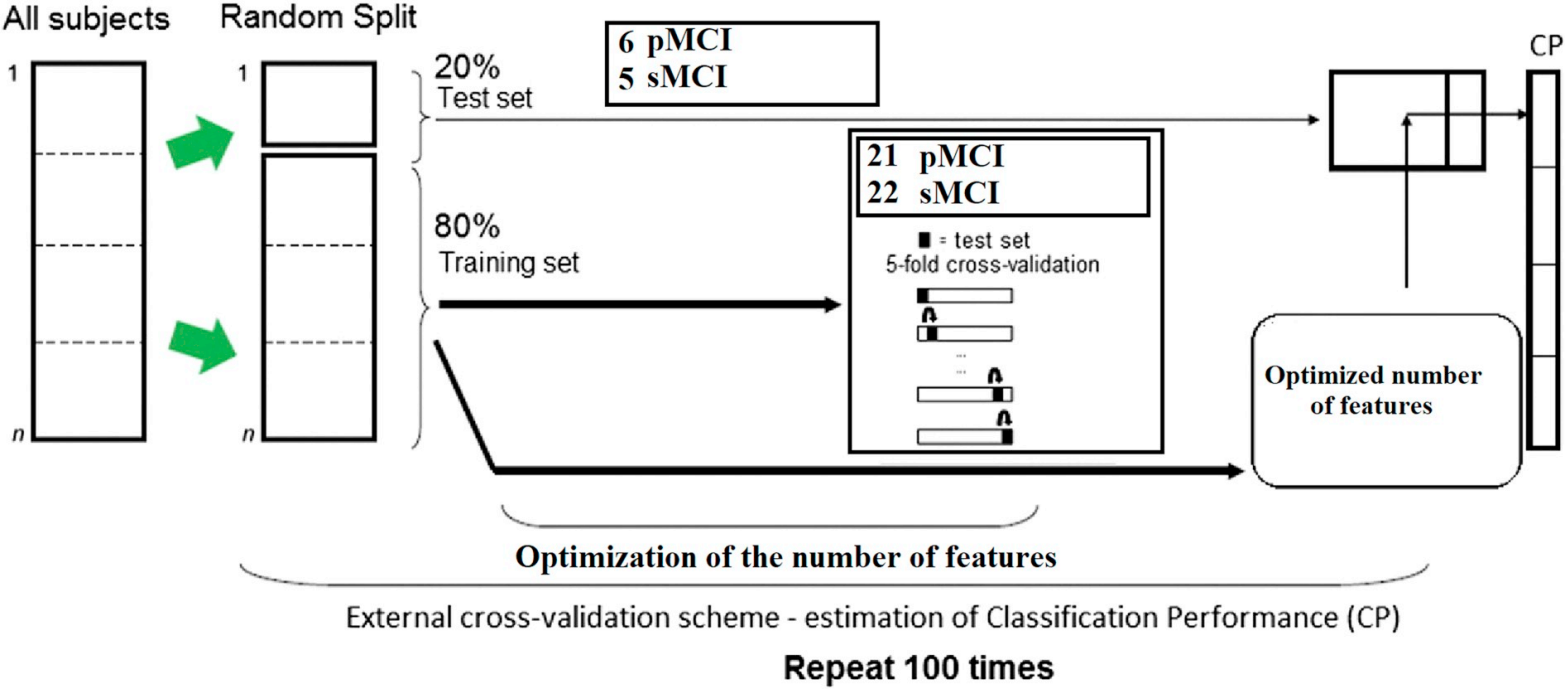
Schematic illustration of the adopted CV scheme.

We estimated the temporal distance between the functional strength time series representing the first and second MEG (Fig. 1D) for every pair of ROIs with the DTW distance metric. The whole analysis was repeated for every subject and frequency bands leading to a high number of potential candidate features. The pair-wise DTW associations of every possible pair of ROIs (*n* = 90*(90–1)/2 = 4005 DTW features) were tabulated in the upper triangular of a matrix with dimensions 90 (ROIs) ×90 (ROIs) (Fig. 1E). The total number of features is 5 (frequency bands) ×4005 DTW features = 20.025 features per subject. This pool of features entered in the machine learning scheme adopted here as a binary classification performance of sMCI vs pMCI subjects. The feature pool was normalized within every fold and independently for every training/test set of features by subtracting the mean and dividing by the standard deviation of every feature estimated from the training set. We ran 5-fold cross-validation (CV) scheme where at every fold, 80% of the subjects (training set) entered the 5-fold CV scheme where we adopted a multi-cluster feature selection (MCFS) algorithm to rank our features and select the set that maximizes the classification performance. Then, we picked up the set of features consistently selected across the 5-folds to train the classifier based on the 80% of subjects and tested in the rest of 20% (test set). The whole procedure was repeated 100 times and independently for every frequency band (Fig. 2). Finally, we aggregated the selected features across the frequencies to design a multiplex biomarker that can potentially predict the converted subjects. Here, we employed a Support Vector Machine (SVM) classifier with Radial Basis Function (RBF) kernel.

Το further validate the machine learning part of our study, we applied an unsupervised feature selection out of the cross-validation scheme. This scenario is a complementary approach to our main approach (Roffo et al., 2017).

### 2.6 Correlate functional connectivity features with MMSE

Canonical Correlation Analysis was introduced by (Hotelling, 1936) as a multivariate statistical technique that attempts to find linear relationships between two datasets of variables. The two datasets can be represented as matrices *X*_1_ and *X*_2_, with dimensions *n* × *p*_1_ and *n* × *p*_2_ respectively, where *n* denotes the number of subjects and *p*_1_ and *p*_2_ are the number of variables (e.g., DTW features and MMSE estimates) in set *X*_1_ and *X*_2_, respectively. CCA searches to find the linear transformations of *X*_1_ and *X*_2_ that are maximally correlated with each other:

(1) $$\max_{u,v}q=u^{T}X_{1}^{T}X_{2}v\mathrm{subject to}\;u_{2}^{2}\leq 1,v_{2}^{2}\leq 1\;\mathrm{and}\;P_{1}\left(u\right)\leq c_{1},P_{2}\left(v\right)\leq c_{2}$$

CCA assumes that the columns of *X*_1_ and *X*_2_ are normalized having a mean of zero and standard deviation of one. The vectors *u* and *v*, with dimensions *p*_1_ × 1 and *p*_2_ × 1, respectively, are the *canonical vectors* (or weights); the vectors *X*_1_*u* and *X*_2_*v*, with dimensions *n* × 1, are the *canonical variables*; and *q* is called the *canonical correlation*.

CCA analysis will be applied to multi-frequency DTW features (30 features) and the delta difference between MMSE of 1st and 2nd session (dMMSE - 1 feature).

#### 2.6.1 Comparison of PCE's classification performance with bi-variate phase coupling estimators

A basic issue in functional connectivity analysis because of signal spread is the signal leakage in MEG and EEG source reconstructed activity. In both EEG and MEG, a spatially widely group of sensors detects the brain activity derived from a single neuronal source. Any correlation between signals estimated at two spatially distance sensors do not necessarily reflect the interaction of two distinct cortical sources. On the contrary, at the sensor level, the same sensor can collect signal from multiple neuronal sources. Therefore, two instantaneously interacting sources at i.e. zero-phase lag are difficult to be distinguished from a single source whose activity recorded by the same sensors (Palva et al., 2018; Wang et al., 2018).

In recent years, novel innovative measures have been introduced to avoid false positive observations of coupling that can be attributed to signal spread. These are: Orthogonalized correlation coefficient (oCC) (Brookes et al., 2012; Hipp et al., 2012), Imaginary part of coherency (ImC) (Nolte et al., 2004), Phase-lag index (PLI) (Stam et al., 2007), Weighted phase lag index (wPLI) (Vinck et al., 2011) and Imaginary phase locking value (iPLV) (Bruña et al., 2018; Dimitriadis, 2018; Dimitriadis et al., 2018a, Dimitriadis et al., 2018b; Dimitriadis et al., 2017a, Dimitriadis et al., 2017b; Dimitriadis and Salis, 2017).

While these methods can be very useful, they have an important limitation. Ignoring near-zero-lag interaction components makes the interaction estimate insensitive to leakage; and also true near-zero-phase-lag interactions will remain undetected.

The estimated interactions can be driven either by (a) true, (b) artificial or (c) spurious interactions among the reconstructed signals (Palva et al., 2018). True interactions refer to real interactions between neuronal sources at specific spatial locations. Artificial interactions reflect false positives interactions that are caused by real interactions between neuronal sources. ‘Significant’ coupling is caused by signal mixing and cross-talk from dominant sources at distant locations reflecting residual effects of the signal spread at the source level. *Spurious* interactions reflect estimated interactions that are false positives and also result from cross-talk (Palva and Palva, 2012). Spurious or ghost interactions occur when signal spread results in pairs of sources in the vicinity of the actual interacting sources to also display significant coupling. For instance, Wang et al. (2018) proposed ‘hyperedge bundling’ to further correct for secondary leakage.

In the present study, we reported the results of classification performance employing also PLV, iPLV and PLI (see section 1 in the Supplementary material).

#### 2.6.2 Reproducibility of PCE over repeat scan cohorts

To further introduce PCE as a proper multivariate phase coupling estimator in functional neuroimaging, we estimated PCE in a previously published repeat scan MEG cohort (see section 2 in the Supplementary material). We estimated functional connectivity graphs (FCG) with PCE over different widths of temporal windows employing cosine similarity as a proper index to quantify the similarity of FCG between the two cohorts.

#### 2.6.3 Quantifying the effect of ghost interactions via a high-order FCG (HO-FCG)

We introduced here a way of quantifying the potential leakage of dynamic functional connectivity analysis via a HO-FCG analysis. Specifically, for every subject, condition and frequency band, we estimated a dynamic functional connectivity graph (dFCG) with size equals to {ROIs × ROIs × epochs}. This dFCG is a low-order graph that tabulates the N = (ROIs × (ROIs − 1))/2 and for ROIs = 90, *N* = 4005 possible pair-wise connectivity estimator over experimental time (epochs). By estimating the cosine similarity between every possible pair of 4005 pairs across the epoch size, we constructed a HO-FCG with size equals to 4005 × 4005. Finally, we estimated the mean cosine similarity of the resulted HO-FCG as an index of how similar the functional strength across the ROIs for each connectivity estimator is (see section 3 in the Supplementary material).

#### 2.6.4 Sensitivity of PCE to zero-lag synchronizations

We explored the sensitivity of PCE to zero-lag synchronization using a Rossler – Lorenz system and different scenarios of volume conduction effect. We compared PCE's performance over PLV and iPLV bivariate phase estimators. Our experiments showed that with the increment of volume conduction effect, PCE demonstrated lower values compared to PLV and higher compared to iPLV. So, PCE encountered both the real and imaginary part of the complex signal. In seems that PCE is less sensitive to volume conduction issues especially to the volume conduction of 0.5 (for further details see section 5.C in the Supplementary material).

#### 2.6.5 Correlation between functional strength and signal power

We estimated the relative signal power for every temporal segment across ROIs, epochs, conditions and frequency bands using fast fourier transform. Complementary, we estimated the functional strength per ROI, condition and frequency band by summing the functional pairwise strengths between every ROI and the rest of 90–1 = 89 ROIs. Practically, we summed the functional strength of every row in the matrix layout of a FCG. Finally, we estimated the absolute correlation between relative signal power and functional strength for every ROI, frequency band and condition across the dimension of epochs. Then, the absolute correlation values were subgroup averaged across ROIs first and secondly across subjects for every condition and frequency band (see section 4 in the supplementary material). We assessed statistical group and condition-based statistically significant differences between group-averaged absolute correlation values by adopting Wilcoxon Rank-Sum Test (*p* < .05, Bonferroni corrected, *p*′ < *p*/6 across frequency bands).

## 3 Results

### 3.1 Classification performance

Table 2 summarizes the evaluation of the proposed feature extraction, selection and classification procedure for the classification of sMCI versus pMCI subjects. The highest classification performance (CP) was succeeded in γ frequency while in the multiplexity scenario; an absolute accuracy (100%) was observed for CENT. All the frequencies performed well in the prediction of sMCI versus pMCI. Employing the unsupervised feature selection approach, we succeeded to absolute discriminate the two groups.

**Table 2:** Evaluation of classification performance using DTW values in every frequency band and in the multiplexity scenario in both PCA and CENT methods.

	PCA			CENT		
	Classification Performance	Sensitivity	Specificity	Classification Performance	Sensitivity	Specificity
δ (8 features)	0.83 ± 0.01	0.85 ± 0.01	0.87 ± 0.01	0.88 ± 0.01	0.88 ± 0.01	0.89 ± 0.01
θ (10 features)	0.82 ± 0.02	0.81 ± 0.02	0.80 ± 0.01	0.87 ± 0.02	0.85 ± 0.02	0.88 ± 0.01
α (9 features)	0.76 ± 0.01	0.72 ± 0.02	0.78 ± 0.01	0.79 ± 0.01	0.7 ± 0.02	0.81 ± 0.01
β (10 features)	0.82 ± 0.02	0.81 ± 0.02	0.80 ± 0.02	0.87 ± 0.02	0.88 ± 0.02	0.85 ± 0.02
γ (12 features)	0.91 ± 0.02	0.87 ± 0.02	0.86 ± 0.00	0.94 ± 0.02	0.88 ± 0.02	1.00 ± 0.00
δ + θ + α + β + γ (7 + 4 + 11 + 2 + 6 features)	0.96 ± 0.00	0.92 ± 0.00	0.93 ± 0.00	1.00 ± 0.00	1.00 ± 0.00	1.00 ± 0.00

### 3.2 The topology of the selected features

**Fig. 3:**
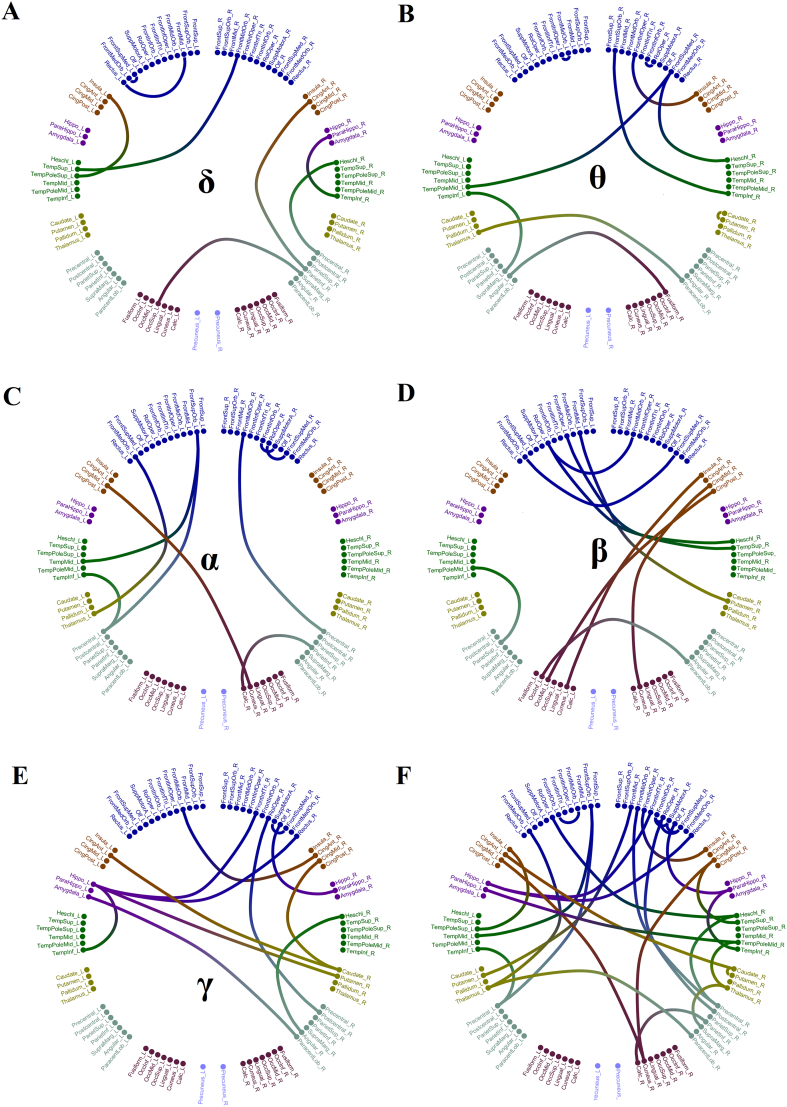
A visualization of the selected frequency-dependent connections using a circular representation of the 90 anatomical ROIs. The left semi-circle represents the 45 ROIs of the left hemisphere while the right semi-circle the homolog ROIs of the right hemisphere. We colored differently the ROIs that belong to a sub-network. Topological layouts of the selected DTW features for each frequency band and in the multiplex scenario. A. 8 features for δ. B. 10 features for θ. C. 9 features for α. D. 10 features for β. E. 12 features for γ. F.30 features for the multiplex integrated approach.

**Fig. 4:**
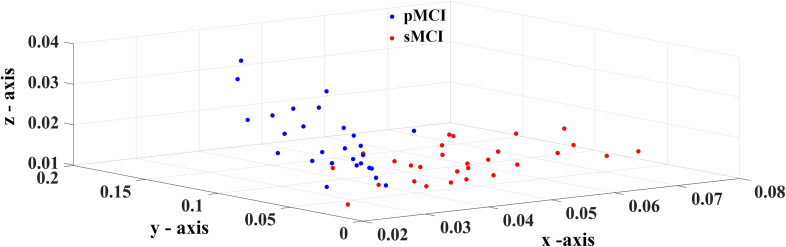
Illustration of both groups in a 3D plot using the three most discriminative features. Every colored dot corresponds to a single subject. One can clearly see the tendency of a clear linear discrimination of both groups. X-axis: Right precentral – Right Heschl's gyrus from δ. Y-axis: Right Caudate Nucleus – Right Putamen from θ. Z-axis: Left Left midcingulate – Right Cuneus from α.

**Fig. 5:**
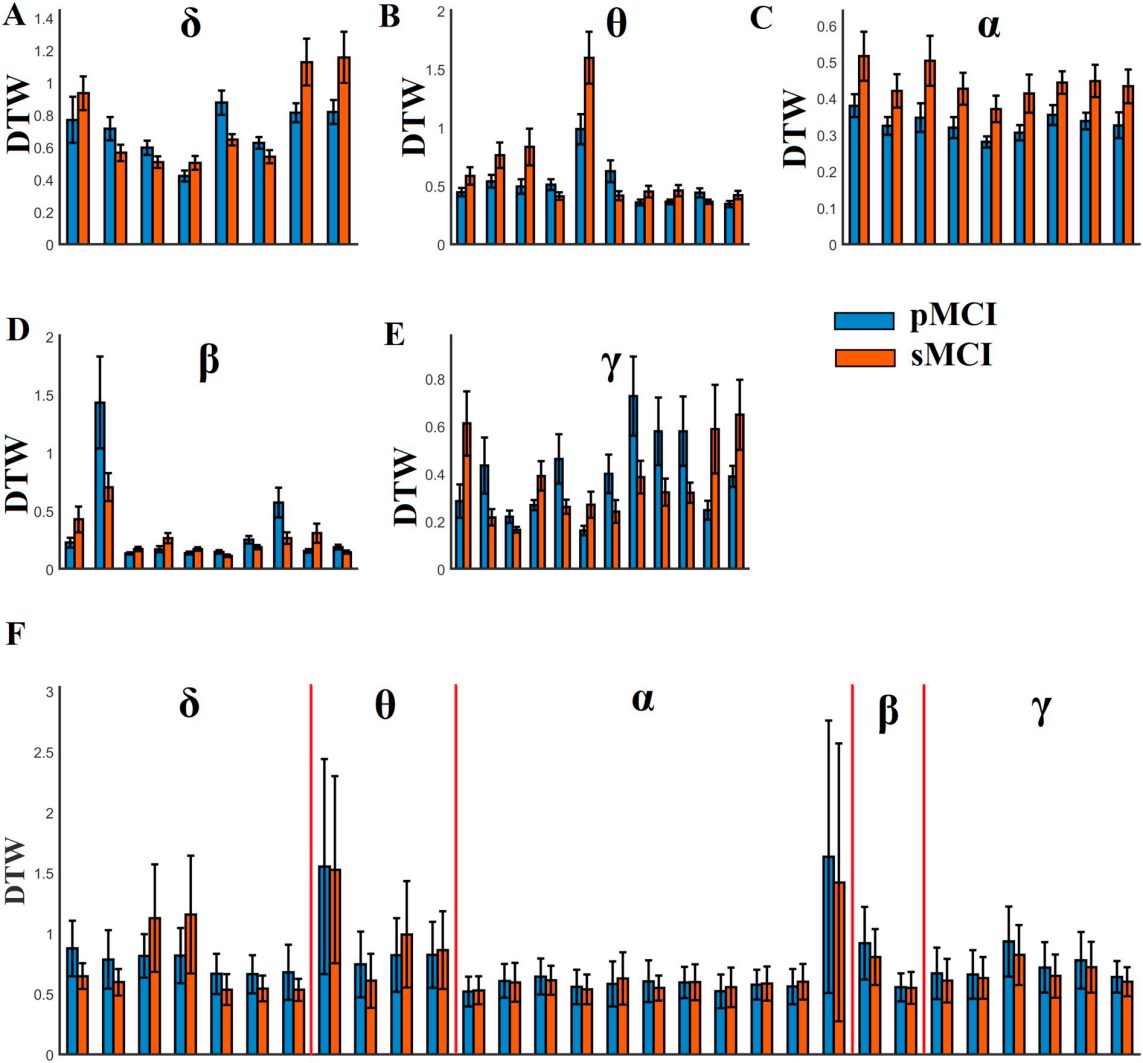
Group-averaged DTW values for the selected features demonstrated topologically in Fig. 3 in both groups and every frequency band. A. δ frequency band. B. θ frequency band. C. α frequency band. D. β frequency band. E. γ frequency band. F. multi-frequency/multiple scenario. Vetical lines separate the subset of frequency dependent DTW features.

We demonstrated the selected features-connections derived from the proposed methodology. Fig. 3 illustrates the network topology of the selected connections across the five frequency bands adopting a circular network layout. Demonstration of the 54 subjects in a 3D plot is given in Fig. 4. We employed the three most discriminative features selected with both the supervised and unsupervised approaches. It is clear the tendency of a clear discrimination of the two groups. Fig. 5 shows the group-averaged DTW values of the selected features. One can see the mixture of higher – lower mean DTW values for pMCI compared to sMCI. However, in α and also in θ frequency band there is a consistent pattern of significant lower DTW values in pMCI compared to sMCI.

**Fig. 6:**
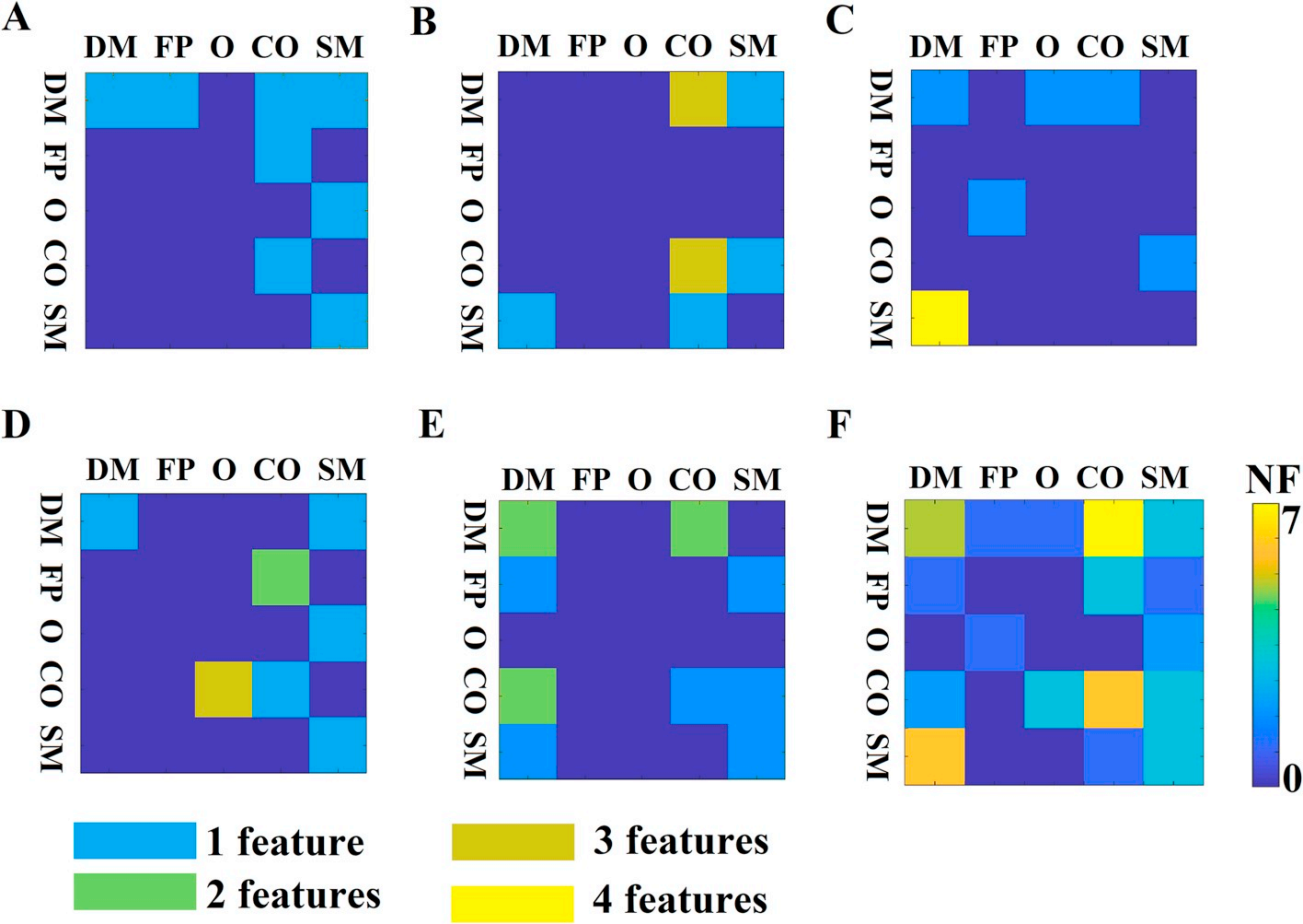
Summarization of the selected features within and between well-known brain networks. A–E) From δ to γ, a representative 2D mapping of the selected features within and between five brain networks. F) We aggregated the selected features across the five frequency bands shown in A-E). (DMN: default mode network, FP: fronto-parietal, O: Occipital, CO: Cingulo-opercular, SM: sensorimotor).

Figure 6 A–E demonstrates the distribution of the selected connections within and between five brain networks across the frequency bands. Fig. 6F tabulates the aggregation of the selected features across the frequency bands. The analysis revealed most of the selected connections are located between ROIs between DMN-CO, within the DMN, within CO and between DMN-SM (24 out of 49 features).

### 3.3 Modeling MMSE changes with the selected DTW features

**Fig. 7:**
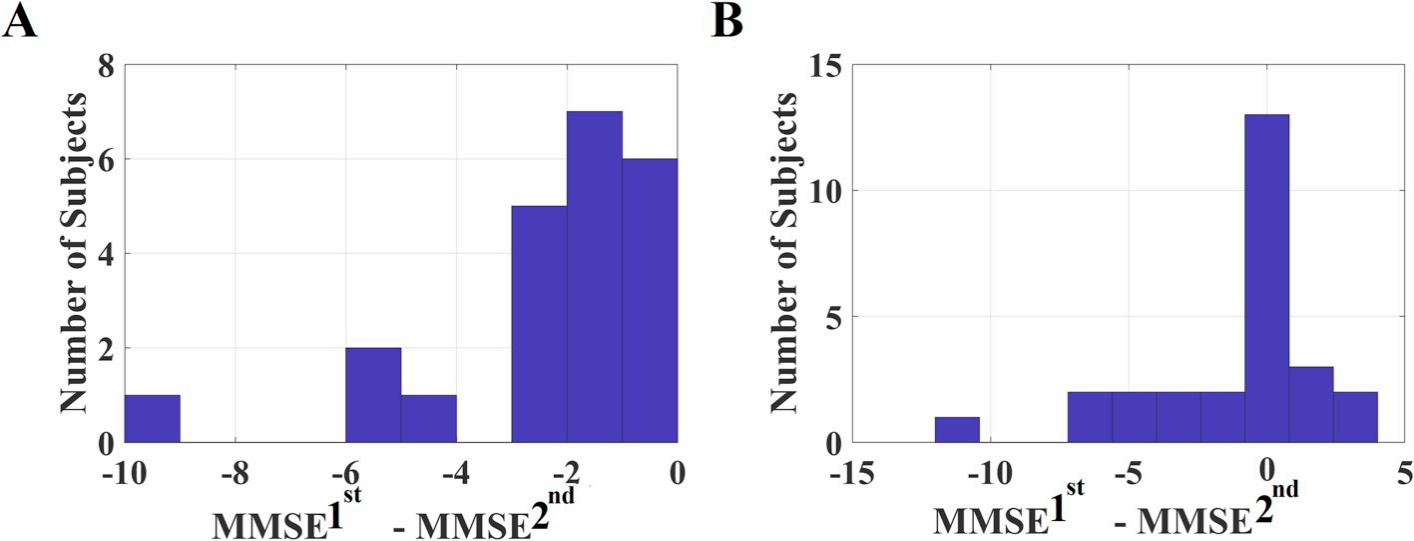
Delta difference of MMSE^1stMEG^ – MMSE^2ndMEG^ in: A) pMCI group and B) sMCI group.

**Fig. 8:**
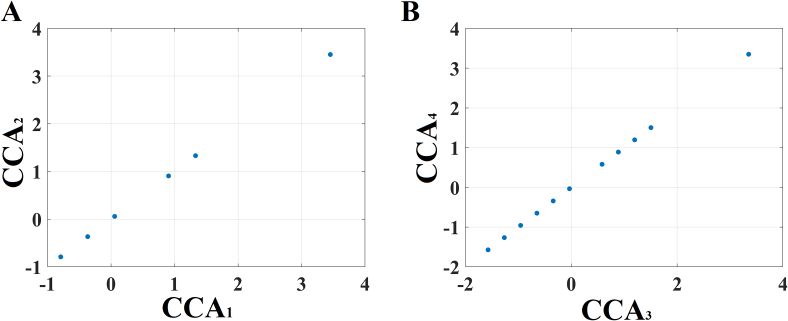
CCA analysis of DTW selected features with delta difference of MMSE^1stMEG^ – MMSE^2ndMEG^ in: A) pMCI group and B) sMCI group.

In an attempt to link differences of MMSE between the first and the second MEG with DTW features, we adopted canonical correlation analysis (CCA) (Hotelling, 1936). We first estimated the delta difference of MMSE^1stMEG^ – MMSE^2ndMEG^ while the whole analysis was repeated twice independently for each group. CCA analysis was performed on 22 pMCI and 27 sMCI subjects. Fig. 7 demonstrates the delta difference of MMSE^1stMEG^ − MMSE^2ndMEG^ for pMCI and sMCI. Fig. 8 plots the canonical variables independently for the two groups.

The model in both groups comprises 4 vectors:

• *U*_*1*_, a {22 for pMCI, 27 for sMCI} × 1 vector of individual subject weights derived from the multi-frequency DTW set of features (in which each value describes the extent to which a given subject is positively or negatively correlated with this mode of population variation with respect to DTW values)
• *V*_*1*_, a {22 for pMCI, 27 for sMCI} × 1 vector, of individual subject weights, derived from the delta difference of MMSE scores between pre and post condition (and which is highly correlated with *U*_*1*_*, r* = 0.87)
• *A*_*1*_, a 1 value of CCA mode weight relating to the 1 component related to delta difference of MMSE scores between pre and post condition fed into the CCA (i.e., the extent to which combinations of delta difference of MMSE scores between pre and post condition relate to mode weights-vector *U*_*1*_)
• *B*_*1*_, a {30 multi-frequency DTW set of features} × 1 vector describing the extent to which each DTW value relates to mode weights-vector *V*_*1*_.

The *p*-value of the Chi-Square test for both CCA was *p* = .00014 (*r* = 0.987) and *p* = .00031 (*r* = 0.968) for pMCI and sMCI, correspondingly.

For the pMCI group, the analytic equations of the two canonical variables are:

CC1=−0.42×dMMSE andCC2=−1.2710×DTWδ1+1.2629×DTWδ2−0.3563×DTWδ5+4.2247×DTWδ7−3.8733×DTWδ8−3.6440×DTWθ2+4.4173DTWθ3−0.0752xDTWθ5+0.4051xDTWθ6+1.6068xDTWθ7−0.0517xDTWβ1−0.0678xDTWβ2+2.728xDTWβ8−0.5383×DTWγ1+5.0264×DTWγ2−1.8760×DTWγ5−0.6674×DTWγ7−0.1785×DTWγ8+1.1694×DTWγ9−0.8150×DTWγ10+5.3895×DTWγ12For the sMCI group, the analytic equations of the two canonical variables are:

CC3=−0.30×dMMSE andCC4=−0.8103×DTWδ1+2.0579×DTWδ2+0.2951×DTWδ7−1.4606×DTWδ85.4130×DTWθ1−2.0202×DTWθ2+0.5917DTWθ3+0.1935xDTWθ5−8.1649xDTWθ73.3325xDTWa1+5.1563xDTWa3−3.6733xDTWa6−8.3304xDTWa7+0.8522xDTWa82.0028xDTWβ1−0.7141xDTWβ2−8.0271xDTWβ8−2.5178xDTWβ92.4710×DTWγ1+3.1583×DTWγ4+4.7490×DTWγ6−4.8469×DTWγ7+5.5724×DTWγ8−0.1337×DTWγ11+0.9710×DTWγ12

CCA revealed characteristic sub-groups within both groups.

In Fig. 8A, one can see that there are six sub-groups for the 22 pMCI and eleven sub-groups for the 27 sMCI.

**Fig. 9:**
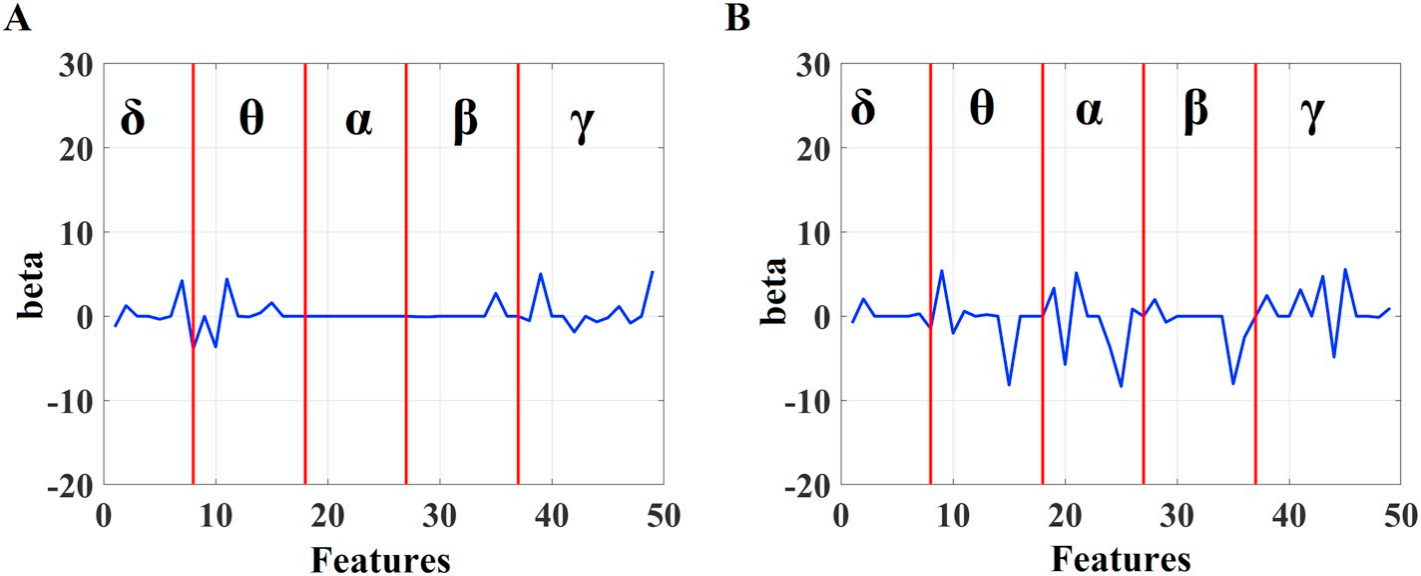
Beta values derived from CCA between delta differences of MMSE versus DTW features. Red vertical lines dissociate the beta values related to frequency-dependent features in: A) pMCI group and B) sMCI group.

Fig. 9 illustrates the b-values of the 49 multi-frequency features related to CC_3_ and CC_4_ in a) pMCI and b) sMCI groups. One can clearly see that the contribution of α and β frequency in CCA for the pMCI group is negligible compared to sMCI.

### 3.4 PCE outperformed bivariate phase estimators

PCE overcame the three adopted bivariate phase connectivity estimators based on classification performance (see section 1 and STable 1 in the Supplementary material). A consistent observation is that definition of representative virtual time series per ROI plays a key role in the classification performance where CENT overcame PCA across the four connectivity estimators.

### 3.5 High reproducibility of PCE in a repeat scan scenario

S1 illustrates the group-averaged cosine similarity of PCE between FCGs derived from the two scan sessions over the studying of the frequency bands. Our results revealed a high reproducibility of functional connectivity patterns based on PCE supported by low cosine similarity index (< 0.1; see section 2 in the Supplementary material).

### 3.6 Quantify a potential effect of ghost interactions via a HO-FCG approach

S3 demonstrates the group-averaged mean cosine similarity index derived from a HO-FCG for every connectivity estimator, frequency band and for both PCA-CENT methods (see section 3 in the Supplementary material). Following a statistical analysis, we revealed that PCE demonstrated the lowest mean cosine similarity value across frequency bands and in both PCA-CENT. Interestingly, the mean cosine values were consistent across frequency bands and in both PCA-CENT methods for every connectivity estimator.

### 3.7 Sensitivity of PCE to zero-lag synchronizations

We explored the sensitivity of PCE to zero-lag synchronization using a Rossler – Lorenz system and different scenarios of volume conduction effect. We compared PCE's performance over PLV and iPLV bivariate phase estimators over various coupling strength and volume conduction/spurious level effects.

Under this assumption, volume conduction/source leakage occurs with zero-lag propagation. In other words, the phase difference of the part of the signals related to such spurious connectivity must be zero.

(For further details see section 5 in the Supplementary material).

### 3.8 Correlation of functional strength and signal power

S4 illustrates the subgroup-averaged absolute correlation values for each condition and frequency band (see section 4 in in the Supplementary material). The subgroup-averaged absolute correlation values were below 0.3 with high variability where we didn't reveal any interesting pattern or any group-difference across the frequency bands.

## 4 Discussion

In the current work, we presented a multivariate functional connectivity approach to investigate its ability to predict the progressing from MCI to AD using a CV and SVM classifiers. First, we computed the PCE to study the distribution of phase differences from the 90 ROIs. Then, we built a dFCG for each subject and frequency band with the aim to estimate the temporal distance between the two times series and to observe the FC changes over time of each pair of ROIs between the first and second MEG session by means of the DTW. Finally, based on the DTW matrices, we obtained a pool of features for each frequency band that entered in a 5-fold CV classifier, where a MCFS algorithm helped to rank and select the set of features for classification. Additionally, a SVM with RBF kernel was used under a multi-layer, multi-frequency scenario to design a multiplex biomarker in order to predict the conversion from MCI to AD. Our results revealed a better performance for CENT compared to PCA approach while PCE outperformed highly used bivariate phase connectivity estimators (see Supplementary material).

Thus, we found that all frequency bands on its own succeeded and had a good and strong performance in classifying the two groups, being the gamma band (γ) the frequency with the highest accuracy in the classification (94%). Importantly, when we integrated in a multi-layer scenario, the multi-frequency DTW features, we obtained a classification performance of 100% to discriminate between sMCI and pMCI. We obtained an absolute accuracy with the CENT method compared to the PCA. Although it is known that such a perfect accuracy is not necessary related to the predictive power of the model (Valverde-Albacete and Peláez-Moreno, 2014), this result clearly suggest that in our sample there are salient differences in the multivariate PS patterns across the whole frequency spectrum between MCI patients who convert to AD after 3 years and those who not. The SVM technique is able to reveal such differences and use them to separate very efficiently converters from non-converters, which opens the way to its application in the clinical neurology.

A recent study reported an impairment of hippocampus and posterior brain areas in AD using MEG source-reconstructed activity (Yu et al., 2017). They followed a multi-layer approach constructed via the integration of different functional brain networks layers each one represented a frequency-dependent network layer. Here, we showed that following a proper feature selection from the pool of DTW features across the multi-frequency network layers could lead to a better performance compared to single network layers. Additionally, the integration of single-layer features across the frequency bands produced lower classification accuracies which further supported our strategy. However, we will attempt in future studies to integrate both intra and cross-frequency coupling estimates in a single network layer under the framework of dominant intrinsic coupling model (DICM) (Dimitriadis et al., 2018a; Dimitriadis et al., 2017a, Dimitriadis et al., 2017b). It is important to study all possible interactions simultaneously and not isolated as it is highly used till now from the neuroscience community.

Nowadays, there is growing evidence that the first stages of AD are associated with profound functional alterations of brain networks that seem to be structurally largely intact. For example, hippocampal hyperactivity and the disruption of the DMN have been demonstrated in people at genetic risk for AD (Bookheimer et al., 2000; Quiroz et al., 2010) and people with early AD (Dickerson et al., 2005)

It is known that during this early phase of the disease, soluble Aß oligomers and amyloid plaques alter the function of local neuronal circuits and large-scale networks by disrupting the balance of synaptic excitation and inhibition (E/I balance) in the brain. Recently, the analysis of animals models of AD revealed that an Aß-induced change of the E/I balance caused hyperactivity in cortical and hippocampal neurons, a breakdown of slow-wave oscillations, as well as network hypersynchrony (Grienberger et al., 2012), thus suggesting that hyperactivity is one of the earliest dysfunctions in the pathophysiological cascade initiated by abnormal Aß accumulation (Busche and Konnerth, 2016).

Later in the disease this hyperactivity, mainly observed in earlier stages of AD, is followed by a hypoactivity (Francis et al., 1993; Palop and Mucke, 2010), which is characteristic of more advance stages of the disease. Thus, AD has been considered as a “disconnection syndrome”, not only due to the death of neurons and connections, but also to the disruption of functional and structural brain networks (Delbeuck et al., 2003).

Regarding the topology of the features that exhibited the highest classification performance, we found that most of the regions belonged to a small set of brain networks: the DMN, CO, FP and also, frontotemporal networks, whose alteration is related to the loss of neurons and synapses that causes major atrophy and malfunctioning of those brain areas that are usually affected in AD, such as temporal gyrus, parietal lobe, and parts of the frontal cortex and cingulate gyrus (Li et al., 2018). As a result of this brain degeneration, AD has been considered as a disconnection syndrome (Delbeuck et al., 2003) as mentioned earlier. Our results revealed the involvement of the DMN as a feature affected by the course of the disease over time. It is well known that this network is usually disrupted during the continuum of AD (Canuet et al., 2015; Garcés et al., 2014; Mevel et al., 2011; Wu et al., 2011), due to the amyloid deposition (Buckner et al., 2009; Sheline et al., 2010). Additionally, we found the engagement of the frontoparietal and cingulo-opercular networks in the classification between sMCI and pMCI. These networks are responsible for coordinating and controlling the executive functions in the brain, which are typically impaired in AD and MCI patients (Lafleche and Albert, 1995; Perry and Hodges, 1999). Lastly, the frontotemporal network resulted in a feature from the classification analysis is usually affected by the course of the disease and related to the common memory impairments found in AD (Buckner, 2004). Additionally, several studies reported that the interaction between these executive networks and the DMN is essential for performing complex cognitive tasks, being considered as a marker of cognitive health (Fox et al., 2005; Spreng et al., 2010).

There is a significant growing interest in detecting early markers of AD pathology linked with alterations of brain functionality in the very early stages of the disorder. Previous studies explored static connectivity analysis on the source level in subjective cognitive decline (SCD) subjects (López-Sanz et al., 2016) and also for the first time in healthy controls, SCD and MCI subjects (López-Sanz et al., 2017) targeting to alpha frequency band. They revealed aberrant functional connections in both SCD and MCI compared to healthy controls validating the sensitivity of MEG source connectivity to detect the preclinical pathology of human brain dynamics. Here, we analyzed dynamic source functional connectivity networks in pre and post condition aiming to define a connectomic biomarker that can differentiate the stable from progressive MCI patients. We adopted DTW as a proper distance measure between two time-series here that represent the fluctuations of functional connectivity strength between two ROIs in pre and post condition. Higher values of DTW can be interpreted as a temporally decoupled index while lower values as a temporal coupled index. Here, in the single-frequency (layer) approach, we revealed higher DTW values for pMCI compared to sMCI in theta and alpha frequencies while the rest of frequencies demonstrating a mixed behaviour (Fig. 5). The topology of these selected DTW-based features is shown in Fig. 3. López-Sanz et al. (2017)) showed lower functional strength for SCD and MCI in alpha band. Here, we additionally revealed a temporally asynchronous behaviour of dynamic functional connectivity in theta and alpha, two frequency bands related to cognitive and memory performance (Klimesch, 1999; Moretti, 2015).

The topology of the multi-frequency (layer) DTW features showed in Fig. 3F and tabulated explicitly in Table 3 involves a spatial distributed network. Particularly, most of the connections involve the frontal brain areas, parietal, thalamus, (para) hippocampal and supplementary motor brain areas. From the 30 features, only 3 included interhemispheric links and the rest were equally classified as intrahemispheric in both hemispheres. A recent fMRI study observed connectivity differences between late MCI and early MCI in regions including the frontal lobe regions (medial frontal gyrus, precentral gyrus, postcentral gyrus), temporal lobe regions (superior temporal gyrus, middle temporal gyrus, frontal gyrus, hippocampus), and thalamus (Cai et al., 2015).

**Table 3:** Brain connections (pair of regions) described in Fig. 3. A–E. (*) indicates interhemispheric links.

**δ**	**θ**	**α**	**β**	**γ**
FrontMid_L & Rectus_L	FrontMidOrb_L & FrontMid_L	FrontSupOrb_L & TempMid_L	RolOper_L & FrontMidOrb_R (*)	FrontMidOrb_L & CingAnt_R (*)
Insula_L & TempPoleSup_L	TempPoleMid_L & FrontSupMed_R (*)	FrontSupOrb_L & Precentral_L	RolOper_L & Heschl_R (*)	CingAnt_R & Caudate_R
TempSup_L & FrontMidOrb_L	TempInf_L & SupraMarg_L	FrontMedOrb_L & Thalamus_L	FrontInfOper_L & TempSup_R (*)	Hippo_L & FrontInfOrb_R (*)
OccSup_L & SupraMarg_L	Thalamus_L & ParacentLob_R (*)	CingMid_L & Cuneus_R (*)	FrontMid_L & Putamen_R (*)	Amygdala_L & ParacentLob_R (*)
FrontSupMed_L & FrontMedOrb_L	Angular_L & Fusiform_R (*)	TempInf_L & Precentral_L	FrontMedOrb_L & FrontSupMed_R (*)	Hippo_L & TempInf_L
CingAnt_R & ParietInf_R	Caudate_R & Putamen_R	Calc_R & ParietInf_R	TempPoleMid_L & ParietSup_L	Angular_R & Heschl_R
ParaHippo_R & TempInf_R	FrontSupOrb_R & TempInf_R	FrontMidOrb_R & Precentral_R	OccInf_L & ParacentLob_R (*)	RolOper_R & Postcentral_R
Precentral_R & Heschl_R	FrontInfOper_R & Insula_R	FrontInfOrb_R & SuppMotorA_R	OccMid_L & CingPost_R (*)	SuppMotorA_R & FrontSupMed_R
	FrontInfOrb_R & RolOper_R	RolOper_R & FrontSupMed_R	Cuneus_L & Insula_R (*)	Olf_R & ParaHippo_R
	FrontSupMed_R & Heschl_R		Cuneus_R & CingMid_R	CingMid_R & Caudate_R


**δ+θ+α+β+γ**	
Insula_L & TempPoleSup_L	Thalamus_R & SupraMarg_R
CingAnt_R & Caudate_R	Caudate_R & Putamen_R
CingMid_L & Cuneus_R	Thalamus_R & TempPoleMid_R
Hippo_L & FrontInfTri_R	ParaHippo_R & TempInf_R
Hippo_L & Rectus_R (*)	CingMId_R & TempSup_R
ParaHippo_L & TempPoleMid_R (*)	Postcentral_R & RolOper_R
TempMid_L & FrontSupOrb_L	Precentral_R & FrontalMidOrb_R
TempInf_L & Precentral_L	Precentral_R & Heschl_R
FrontSupOrb_L & Precentral_L	RolOper_L & Heschl_R (*)
Calc_R & ParietInf_R	Olf_R & ParaHippo_R
Cuneus_R & CingMid_R	FrontInfOper_R & Insula_R
Thalamus_L & ParacentLob_R (*)	SuppMotorA_R & FrontSupMed_R
FrontMid_R & Putamen_L (*)	FrontInfOrb_R & SuppMotorA_R
FrontMedOrb_L & Thalamus_L	RolOper_R & FrontSupMed_R
Cuneus_R & CingMid_L	FrontMidOrb_L & FrontInfOrb_L

We revealed an interesting subset of DTW-features in pairs of ROIs between the thalamus and other parts of the brain. These thalamo-related networks include thalamo-frontal, thalamo-parietal, thalamo-temporal and thalamo-DMN subnetworks. The decreased temporally decoupled functional connectivity expressed with higher DTW values for pMCI compared to sMCI between the thalamus and the aforementioned brain areas might suggest reduced functional integrity of thalamo-related networks and increased temporally coupled functional connectivity (lower DTW values) indicated that pMCI patients could use additional brain resources to compensate for the loss of cognitive function (Fig. 3F & Table 3).

The olfactory system is a well-defined network that has been implicated in early stages of the AD, marked by impairment in olfaction and the presence of pathological hallmarks of the AD. There are outputs from the olfactory system that reach the parahippocampal region of the brain, including the perirhinal, parahippocampal, and entorhinal cortices (Eichenbauma, 1998). The primary olfactory cortex has connections to brain regions, such as the hippocampus (Haberly, 2001) which is altered in AD. Overall, the olfactory system has substantial connections to areas of the brain that are related to memory and display AD pathology (Franks et al., 2015). Here, we revealed higher DTW values between olfactory and parahippocampal brain areas in pMCI compared to sMCI.

Functional brain connectivity can be quantified with a large number of techniques that can be separated into model-based and data-driven techniques. A famous technique that has been used widely in the literature is the PLV (Lachaux et al., 1999), which is a data-driven connectivity estimator that can capture non-linear interactions between pairs of brain signals. Here, for the first time in MEG and especially in the study of MCI, we adopted a multivariate extension of PLV, the so-called PCE (Cadieu and Koepsell, 2010). There is a big interest in extending bivariate functional connectivity estimators that quantify the interdependence between two time series to its multivariate extension (Pereda et al., 2005). Pair-wise connectivity analysis is more sensitive to spurious correlations, particularly in those cases where one driver lead two responses. In that scenario, both responses may have a common driver even when seem to be completely independent (Sakkalis, 2011). The advantage of PCE estimator is that it is data-driven and does not depend on the reliability of the fitted MVAR (Multivariate Vector Auto-Regressive) model. We reported in a second repeat-scan dataset, the high reproducibility of PCE across experimental time at resting-state. Additionally, PCE outperformed the frequent used bivariate phase connectivity estimators of PLV, PLI and iPLV (see Section 1 in the Supplementary material).

Neuroscience community that works under the umbrella of functional connectivity searched to identify connectivity estimators and techniques to remove zero-phase lag interactions that are spread from the same neuronal sources, which are referred to as primary signal leakage in source reconstructed EEG/MEG data. Moreover, the primary signal leakage may contribute in spurious estimates of functional connectivity between brain areas surrounding two genuinely connected brain regions (Palva and Palva, 2012). Widely used connectivity estimators like oCC (Brookes et al., 2012; Hipp et al., 2012), ImC (Nolte et al., 2004), PLI (Stam et al., 2007), wPLI (Vinck et al., 2011) and iPLV, (Bruña et al., 2018; Dimitriadis, 2018; Dimitriadis et al., 2018a, Dimitriadis et al., 2018b; Dimitriadis et al., 2017a, Dimitriadis et al., 2017b; Dimitriadis and Salis, 2017) are not able to completely eliminate the secondary leakage (Palva et al., 2018; Wang et al., 2018). A recent study (Wang et al., 2018) has developed a novel

approach, called “hyperedge bunding”, to further correct the secondary leakage. We will consider this new method in future studies under the framework of designing robust connectomic biomarkers for MCI. Here, we showed that the adopted multivariate connectivity estimator PCE outperformed bivariate phase estimators in terms of classification performance while it was proved high reproducible. Additionally, it behaves well in zero-lag effects and the adopted HO approach showed lower values of cosine similarity values compared to bivariate phase estimators (see Supplementary material). Following, a volume conduction scenario, we showed that PCE is also insensitive to zero-lag synchronization while it is more sensitive to the real part of the complex signal compared to iPLV.

Intrinsic coupling is a characteristic feature of ongoing brain activity with rich spatiotemporal patterns. There are two intrinsic coupling types: the phase coupling and the correlation of the envelope of the band-limited oscillatory brain signals (Engel et al., 2013). There is a general hypothesis that phase coupling has a loose relationship with structural connectivity while the correlation of the envelope a tighter one. Additionally, phase coupling seems to be more sensitive to aberrant functional connectivity in various brain disorders even in the absence of structural changes (Marzetti et al., 2013). However, more neurophysiological explorations of both correlation of the envelope and phase coupling need to be estimated between virtual source space time series (Brookes et al., 2011; Dimitriadis et al., 2018a, Dimitriadis et al., 2018b; Hipp et al., 2012).

Another novelty of our study is the adaptation of DTW as a proper distance metric to quantify the similarity of the fluctuated dynamic functional phase coupling strength of a pair of brain areas between 1st and 2^nd^ MEG session. In biomedical research, DTW has been mainly used to classify signals into different sub-groups by comparing each one with standard templates (Forestier et al., 2012). Another study first employed DTW as a novel task-based functional connectivity estimator between MEG sensor time series tailored to ERP multichannel recordings (Karamzadeh et al., 2013).

We adopted a well-designed cross-validated machine learning approach that succeeded to extract meaningful DTW features related to (dis)similarity of the temporal fluctuation of the functional coupling between the first and the second MEG recording. Our analysis untangled informative DTW features across the brain areas and the frequency bands studied. We believe that our results are very interesting succeeding to place MEG on the top of the hierarchy of neuroimaging modalities which are sensitive to prodromal stages of AD. Current analysis has basic advantages over previous analytic attempts. First of all, we adopted a multivariate phase coupling estimator instead of a bivariate connectivity estimator. Secondly, a time-varying approach has been followed leading to the construction of time series describing the fluctuation of coupling strength across experimental time and at every frequency band. Finally, machine learning supported the effectiveness of the current strategic analytic pathway.

CCA analysis between the delta difference of MMSE^1stMEG^ – MMSE^2ndMEG^ and the selected DTW values revealed a stronger multi-frequency contribution of DTW values to the MMSE^1stMEG^ – MMSE^2ndMEG^ for sMCI in comparison with pMCI. Especially, the contribution of α and β frequency bands to the CCA for the pMCI group is negligible compared to sMCI one. This result is supported by recent evidence that α disruption starts from subjective cognitive decline stage (López et al., 2016), and finish with those MCI that finally progressed to AD (Lopez et al., 2014b). Moreover, α and β network disruption has been observed in AD (Koelewijn et al., 2017).

Our study presents a data-driven analytic pathway combining source-reconstructed template-oriented brain activity at resting-state, network neuroscience, and machine learning techniques. Our results are very informative in the understanding of the brain alterations occurred in subjects that will progress from MCI to AD. MEG is a neuroimaging modality that the last few years has demonstrated its potentiality to reveal novel and complementary information to functional (fMRI) related to prodromal stages of AD (Dimitriadis et al., 2018a; Koelewijn et al. 2017; López et al. 2016; Peng et al. 2016). We strongly believe that MEG can play a pivotal role in the application of such methodologies in a daily clinical routine practice supported also by its lower cost compared to fMRI.

## Data availability

The data that support the findings of this study are available from the corresponding author upon reasonable request.

## Author contributions

S.P. and S.D. analyzed the data, prepared figures, and wrote the main manuscript text. M.E.L wrote the manuscript. E.P. and F.M. supervised data analyses and wrote the manuscript. All authors read and approved the final manuscript.

## Declaration of Competing Interest

None.

## Appendix A Supplementary data

Supplementary material Image 1
