# Supplementary material for: Aberrant MEG multi-frequency phase temporal synchronization predicts conversion from mild cognitive impairment-to-Alzheimer's disease
Source: Neuroimage Clin. 2019 Aug 8;24:101972. doi: 10.1016/j.nicl.2019.101972 (PMC6745514; doi:10.1016/j.nicl.2019.101972)
Supplement: Supplementary file 1 — Supplementary material [file mmc1.docx]

SUPPLEMENTARY MATERIAL

1. **Classification Performance of Bivariate Phase Coupling Estimators**

We reported classification performance of phase locking value (PLV; Lachaux et al., 1999), imaginary part of phase locking value (iPLV; Dimitriadis et al.,2015) and phase lag index (PLI; Stam et al., 2007). STable 1 tabulates the performance, sensitivity and specificity following the same pre-processing steps as with PCE in the main text. To compare the performance between the three adopted bivariate connectivity estimators with PCE, we adopted the same number of features per frequency band.

**S-Table 1**. Accuracy, Sensitivity and Specificity of PLV, iPLV and PLI connectivity estimators.

1. **Accuracy, Sensitivity and Specificity of PLV**

|  | **PCA** | | | **CENT** | | |
| --- | --- | --- | --- | --- | --- | --- |
|  | **Accuracy** | **Sensitivity** | **Specificity** | **Accuracy** | **Sensitivity** | **Specificity** |
| **δ**  **(8 features)** | 0.64 ±0.01 | 0.66 ± 0.01 | 0.69 ± 0.01 | 0.65 ±0.01 | 0.64 ± 0.01 | 0.67 ± 0.01 |
| **θ**  **(10 features)** | 0.67 ±0.02 | 0.65 ± 0.02 | 0.67 ± 0.01 | 0.66 ±0.02 | 0.66 ± 0.02 | 0.68 ± 0.01 |
| **α**  **(9 features)** | 0.63 ±0.01 | 0.65 ± 0.02 | 0.62 ± 0.01 | 0.67 ±0.01 | 0.64 ± 0.02 | 0.63 ± 0.01 |
| **β**  **(10 features)** | 0.63 ±0.02 | 0.66 ± 0.01 | 0.65 ± 0.01 | 0.66 ±0.02 | 0.65 ± 0.01 | 0.67 ± 0.01 |
| **γ**  **(12 features)** | 0.67 ±0.01 | 0.68 ± 0.02 | 0.67 ± 0.02 | 0.71 ±0.01 | 0.69 ± 0.02 | 0.68 ± 0.02 |
| **δ+θ+α+β+γ**  **(7+4+11+2+6 features)** | 0.76 ±0.02 | 0.75 ± 0.02 | 0.74 ± 0.02 | 0.79 ±0.02 | 0.76 ± 0.01 | 0.75 ± 0.02 |

1. **Accuracy, Sensitivity and Specificity of iPLV**

|  | **PCA** | | | **CENT** | | |
| --- | --- | --- | --- | --- | --- | --- |
|  | **Accuracy** | **Sensitivity** | **Specificity** | **Accuracy** | **Sensitivity** | **Specificity** |
| **δ**  **(8 features)** | 0.65 ±0.01 | 0.65 ± 0.01 | 0.68 ± 0.01 | 0.67 ±0.01 | 0.64 ± 0.01 | 0.67 ± 0.01 |
| **θ**  **(10 features)** | 0.66 ±0.02 | 0.64 ± 0.02 | 0.69 ± 0.01 | 0.68 ±0.02 | 0.66 ± 0.02 | 0.68 ± 0.01 |
| **α**  **(9 features)** | 0.65 ±0.01 | 0.66 ± 0.02 | 0.65 ± 0.01 | 0.71 ±0.01 | 0.68 ± 0.02 | 0.67 ± 0.01 |
| **β**  **(10 features)** | 0.66 ±0.02 | 0.67 ± 0.01 | 0.68 ± 0.01 | 0.74 ±0.02 | 0.70 ± 0.01 | 0.69 ± 0.01 |
| **γ**  **(12 features)** | 0.68 ±0.01 | 0.67 ± 0.02 | 0.67 ± 0.02 | 0.72 ±0.01 | 0.69 ± 0.02 | 0.68 ± 0.02 |
| **δ+θ+α+β+γ**  **(7+4+11+2+6 features)** | 0.80 ±0.02 | 0.77 ± 0.02 | 0.76 ± 0.02 | 0.82 ±0.02 | 0.77 ± 0.01 | 0.78 ± 0.02 |

1. **Accuracy, Sensitivity and Specificity of PLI**

|  | **PCA** | | | **CENT** | | |
| --- | --- | --- | --- | --- | --- | --- |
|  | **Accuracy** | **Sensitivity** | **Specificity** | **Accuracy** | **Sensitivity** | **Specificity** |
| **δ (8 features)** | 0.67 ±0.01 | 0.66 ± 0.01 | 0.68 ± 0.01 | 0.68 ±0.01 | 0.66 ± 0.01 | 0.67 ± 0.01 |
| **θ (10 features)** | 0.68 ±0.02 | 0.66 ± 0.02 | 0.67 ± 0.01 | 0.69 ±0.02 | 0.65 ± 0.02 | 0.67 ± 0.01 |
| **α (9 features)** | 0.65 ±0.01 | 0.67 ± 0.02 | 0.66 ± 0.01 | 0.71 ±0.01 | 0.67 ± 0.02 | 0.68 ± 0.01 |
| **β (10 features)** | 0.72 ±0.02 | 0.69 ± 0.01 | 0.67 ± 0.01 | 0.73 ±0.02 | 0.69 ± 0.01 | 0.67 ± 0.01 |
| **γ (12 features)** | 0.74 ±0.01 | 0.72 ± 0.02 | 0.70 ± 0.00 | 0.74 ±0.01 | 0.70 ± 0.02 | 0.71 ± 0.00 |
| **δ+θ+α+β+γ**  **(7+4+11+2+6 features)** | 0.79 ±0.02 | 0.77 ± 0.02 | 0.76 ± 0.02 | 0.82 ±0.02 | 0.77 ± 0.01 | - 1. ± 0.02 |

1. **Stability and Reliability of Functional Connectivity Patterns based on PCE**

To test the stability and reliability of functional connectivity patterns based on PCE, we adopted a repeat MEG scan cohort that has been previously published. Below, we summarized the cohort while we described the details of the approach in order to estimate the consistency of functional connectivity patterns in repeat scan sessions.

**2.1. Subjects**

40 healthy subjects (age *22.85* ± *3.74* years, 15 women and 25 men) underwent two resting-state MEG sessions (eyes open) with a 1-week test-retest interval. For each participant, scans were scheduled at the same day of the week and same time of the day. The duration of MEG resting-state was 5 mins for every participant. The study was approved by the Ethics Committee of the School of Psychology at Cardiff University, and participants provided informed and written consent.

**2.2 MEG-MRI Recordings**

Whole-head MEG recordings were made using a 275-channel CTF radial gradiometer system. 29 reference channels were recorded for noise cancellation purposes and the primary sensors were analysed as synthetic third-order gradiometers (Vrba and Robinson, 2001). Two or three of the 275 channels were turned off due to excessive sensor noise (depending on time of acquisition). Subjects were seated upright in the magnetically shielded room. To achieve MRI/MEG co-registration, fiduciary markers were placed at fixed distances from three anatomical landmarks identifiable in the subject’s anatomical MRI, and their locations were verified afterwards using high-resolution digital photographs. Head localisation was performed before and after each recording, and a trigger was sent to the acquisition computer at relevant stimulus events. For further details see Dimitriadis et al. (2018a, b).

- 1. **Dynamic Functional Connectivity Analysis based on PCE**

We estimated dynamic functional connectivity graphs per subject and frequency band based on PCE over augmenting width of temporal windows. Practically, we started with a temporal window of size equals to 1 min and we increased its width by 10 sec up to 4 mins leading to a total number of 19 temporal windows. Then, we estimated the cosine similarity of the upper triangular of each quasi-static functional connectivity graphs between the two scan sessions independently per subject and frequency bands. Our analysis revealed a high similarity (low values of cosine similarity) between the functional connectivity graphs derived from the two scan sessions across different widths of temporal windows.


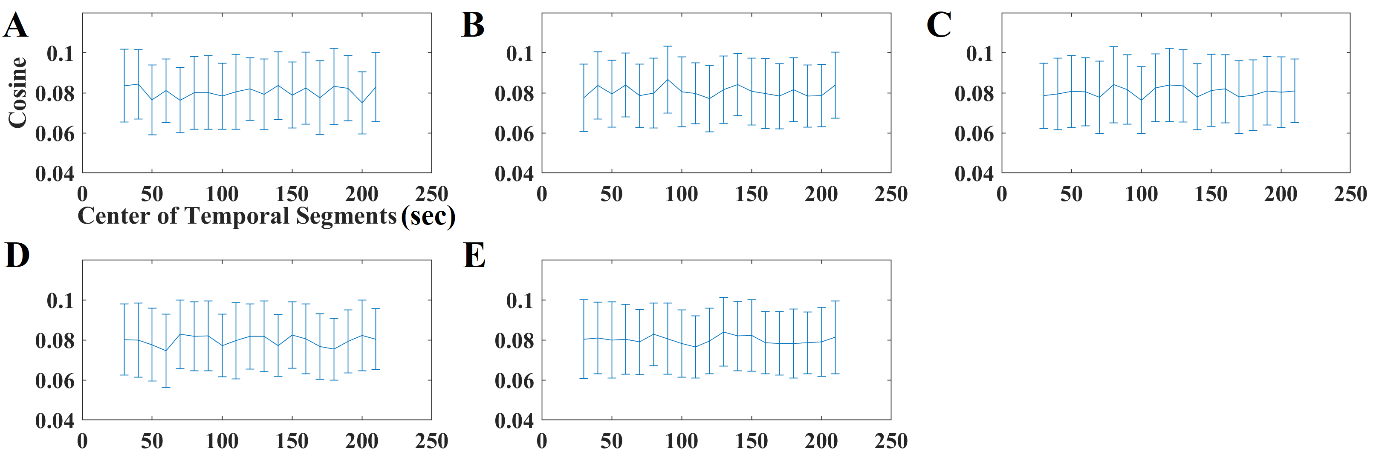


**S1. Group-averaged cosine similarities of functional connectivity graphs between the two scan sessions for each frequency band.**

**Each subplot A – E refers to δ to γ frequency bands**

1. **Quantifying the Effect of Potential Spurious Dynamic Connections via a High – Order Graph Analysis**

In every subject, we estimated a dynamic functional connectivity graph (dFCG) for each frequency band, in pre and post condition and in every connectivity estimator. The size of dFCG is equal to [ ROIs x ROIs x epochs] while the high-order functional connectivity graph (HO-FCG) that estimates the similarity between every possible pair of pairs of ROIs equals to [N x N], where N = (ROIs x (ROIs - 1))/2 and for ROIs = 90, N = 4005.

S2 illustrates an example of a HC subject in δ frequency band in pre and post condition for PCE multivariate connectivity estimator.

S2.A shows the vectorised dynamic functional connectivity patterns of pairs of ROIs for PCE in pre and post conditions for PCA scheme while S4.B demonstrates the cosine of every possible pair of pairs of ROIs across epochs leading to the estimation of a HO-FCG.

S3 illustrates the mean and standard deviation across subjects of the cosine of every possible pair of pairs of ROIs for every frequency band, estimator and in both pre and post condition. They lowest the cosine similarity index the more distinct are the dynamic connectivity patterns between pairs of pairs of ROIs. This index can be seen as a way to encounter the spurious connectivity strength across ROIs. We can clearly see that the group-averaged cosine similarity in HO-FCG called hereafter HO^Cor^ followed a specific pattern across frequency bands and both PCA and CENT schemes. HO^Cor^ characterizes every connectivity estimator independently of the frequency band and schemes that define the characteristic virtual time series per ROI, PCA or CENT.

Following statistical test between connectivity estimators in every frequency band (p< 0.05, Wilcoxon Rank Sum Test, p’<p/6, Bonferroni corrected) revealed that PCE mean cosine similarity index was significantly lower compared to the three bivariate connectivity estimators. This result in conjunction with the classification performance, stability of PCE across time and reliability across repeat scans further support our connectomic biomarker approach.


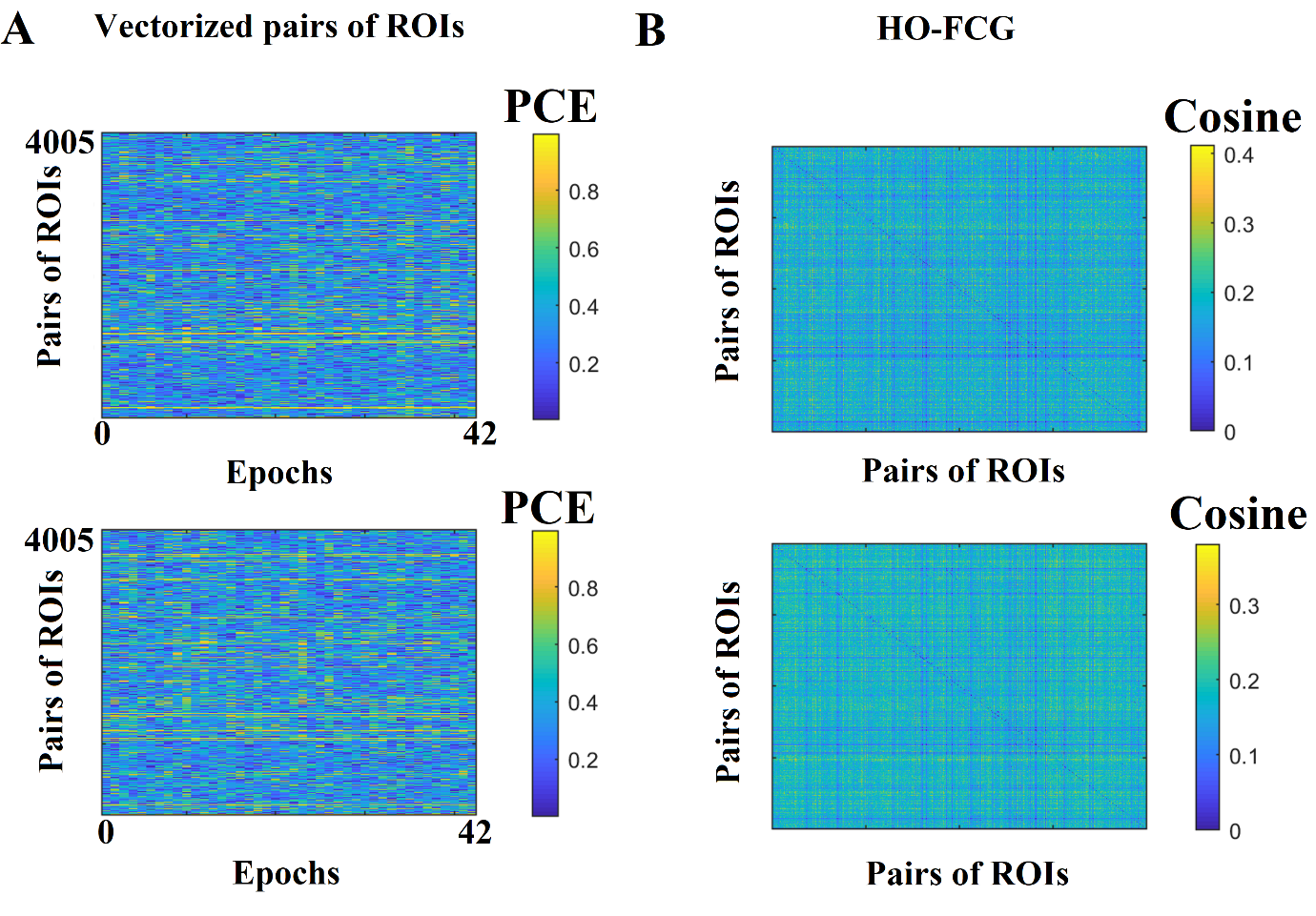


**S2. Vectorised dynamic functional connectivity across pairs of ROIs and the related HO-FCG. An example from a HC subject in δ frequency band for PCE connectivity estimator using PCA scheme.**

1. Vectorization of dynamic functional connectivity strength for every pair of ROIs across experimental time for PCE connectivity estimator in pre and post condition using PCA scheme.
2. Presentation of HO-FCG derived from the pair-wise estimation of cosine similarity from the vectorised dynamic patterns demonstrated in A.


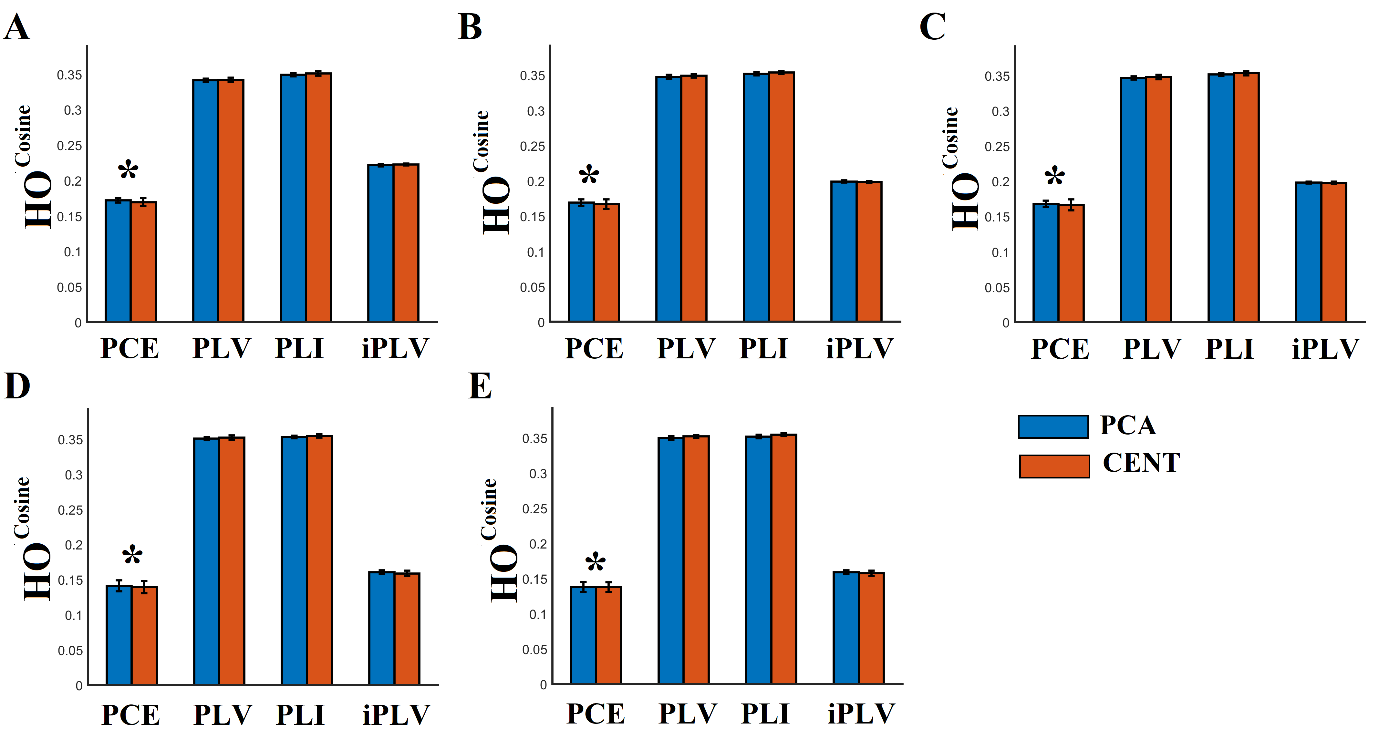


**S3. Group-averaged HO^Cosine^ across subjects in every frequency band, connectivity estimator and in both PCA and CENT schemes.**

1. **Correlation between Signal Spectrum and functional strength across ROIs.**

We quantified the correlation between the signal spectrum and functional strength per ROI, frequency band, subgroup and condition. At first, we estimated the relative signal power per ROI across frequency bands using fast fourier transform. Afterwards, we estimated the functional strength of every ROI by summing the functional weights of each ROI with the rest 89 ROIs (the sum of every row in a matrix that tabulates every functional connectivity graph). Finally, we estimated for every ROI the correlation between relative signal power and functional strength across the number of epochs. Then, the absolute correlation values were averaged across the 90 ROIs and finally across subjects and condition. S4 illustrates the subgroup-averaged absolute correlation values for each condition and frequency bands.


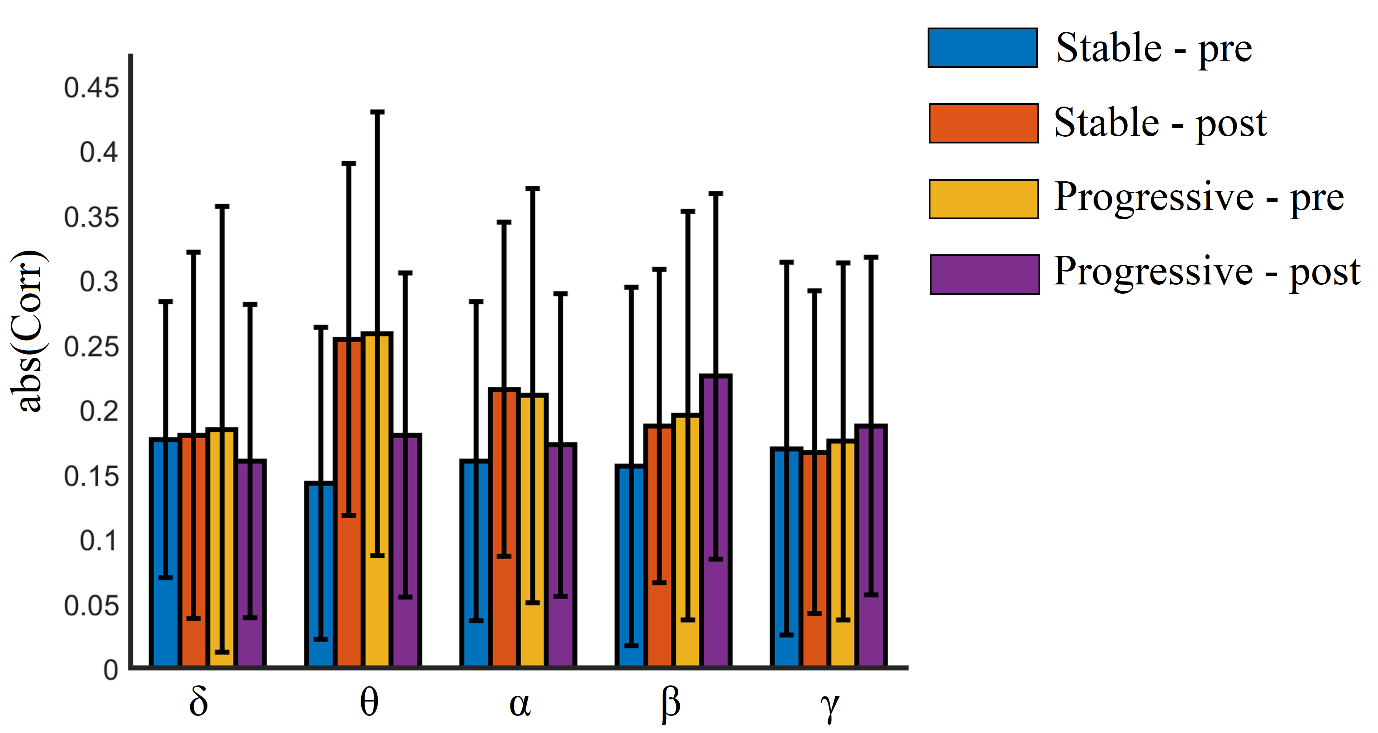


**S4. Sub-group averaged of absolute correlation values between signal spectrum and functional strength across the 90 ROIs.**

**We demonstrated the absolute correlation values independently for each frequency bands, the subgroups and the pre – post conditions.**

1. **Exploring the Sensitivity of PCE in zero lag synchronization**

In order to evaluate the behavior of PCE to zero-lag synchronization compared to PLV, iPLV in signals with a known degree of coupling, we used a pair of coupled chaotic systems: a Rössler system and a Lorenz one (Quiroga et al., 2000). In this setup, the Rössler system acts as driver, and an oscillation frequency can be defined from it. The slave Lorenz system is driven by the Rössler to an extent determined by the coupling parameter C, ranging from zero (completely independent systems) to one. The mathematical definition of the coupled systems is:

**
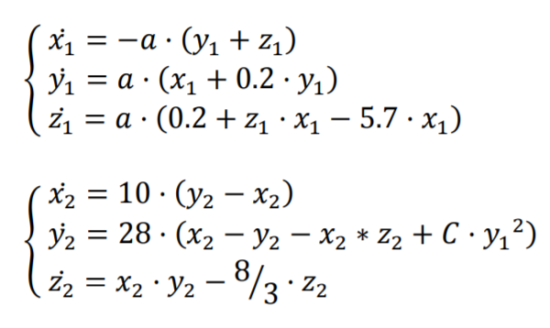
(1)**

where subscripts 1 and 2 refer to the Rössler and the Lorentz system, respectively, is a scaling parameter determining the fundamental frequency of the Rössler oscillator and is the coupling parameter. The scaling parameter was set to 10, establishing the oscillatory frequency at 0.585·π radians per sample, and the oscillatory band was set to 0.570·π to 0.600·π radians per sample. With this setup, we generated 50 pairs of signals of 20,000 samples for different values of C ranging between zero and one (Bruna et al., 2018).

In order to evaluate the behaviour of these PCE and PLV/iPLV bivariate phase connectivity metrics, we used the same pair of chaotic systems defined with Rossler – Lorentz system. The systems show nonzero-lag phase synchronization, and the volume conduction can be introduced using instantaneous linear mixing (Haufe et al., 2013; Porz et al., 2014)

**
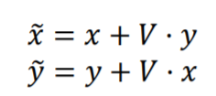
(2)**

where is a parameter determining the amount of mixing. S5 shows the synchronization estimated between the signals described in the previous section using the PLV, iPLV and PCE, and values of 0, 0.1 and 0.5.

Our experiments showed that with the increment of volume conduction effect, PCE demonstrated lower values compared to PLV and higher compared to iPLV. This is because PLV contains only the real part of the complex signal while iPLV only the imaginary part insensitive to zero-lag source leakage. In contrast, PCE seems to be less sensitive to volume conduction issues while it keeps both the real and imaginary part of the complex signal especially to the third case (S5.C).


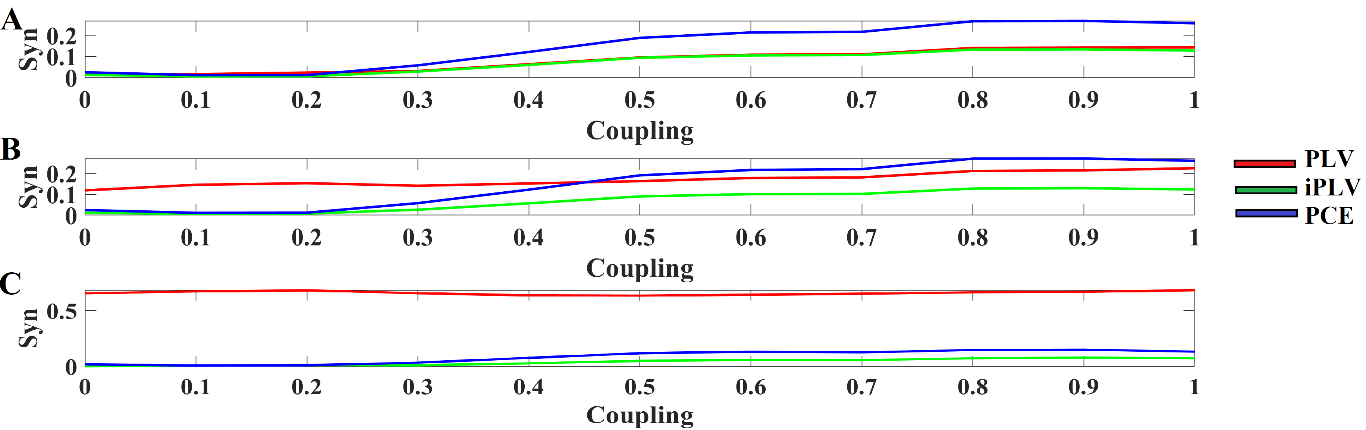


**S5. Values of the synchronization indices analyzed for different couplings for one Rössler and one Lorenz system.**

(a) Synchronization estimated using PLV, iPLV and PCE for non-zero lag scenario.

(b) The same as in (a), after adding a 10% of linear mixing to the signals.

(c) Idem after adding a 50% of linear mixing.

**S-Table2: Regions of the AAL atlas with its respective abbreviation. In the third column, the association of each specific region to the subnetworks of the brain is shown. DMN: Default Mode Network, FPN: Fronto Parietal Network, CO: Cingulo -Opercular Network, O: Visual Network, SM: Sensorimotor Network (Smitha et al., 2017).**

| ***Abbreviation (Right or Left)*** | ***AAL regions*** | ***Subnetworks*** |
| --- | --- | --- |
| Precentral_ | Precentral Gyrus | SM |
| FrontSup_ | Superior Frontal Gyrus | FPN |
| FrontSupOrb_ | Superior Frontal (orbital) Gyrus |  |
| FrontMid_ | Middle Frontal Gyrus | FPN,CO |
| FrontMidOrb_ | Middle Frontal (orbital) Gyrus |  |
| FrontInfOper_ | Inferior Frontal (opercular) Gyrus | CO |
| FrontInfTri_ | Inferior Frontal (triangular) Gyrus |  |
| FrontInfOrb_ | Inferior Frontal (orbital) Gyrus |  |
| RolOper_ | Rolandic operculum | CO |
| SuppMotorA_ | Supplementary Motor Area | SM |
| Olf_ | Olfactory Cortex |  |
| FrontSupMed_ | Medial Frontal Gyrus | DMN,FPN,CO |
| FrontMedOrb_ | Medial Frontal (orbital) Cortex |  |
| Rectus_ | Rectus Gyrus |  |
| Insula_ | Insula | CO |
| CingAnt_ | Anterior Cingulate Gyrus | DMN,CO |
| CingMid_ | Middle Cingulate Gyrus |  |
| CingPost_ | Posterior Cingulate Gyrus | DMN,FPN |
| Hippo_ | Hippocampus | DMN |
| ParaHippo_ | Parahippocampal Gyrus |  |
| Amygdala_ | Amygdala |  |
| Calc_ | Calcarine Sulcus | O |
| Cuneus_ | Cuneus | O |
| Lingual_ | Lingual Gyrus | O |
| OccSup_ | Superior Occipital Gyrus | O |
| OccMid_ | Middle Occipital Gyrus | O |
| OccInf_ | Inferior Occipital Gyrus | O |
| Fusiform_ | Fusiform Gyrus | O |
| Postcentral_ | Postcentral Gyrus | SM |
| ParietSup_ | Superior Parietal Lobule |  |
| ParietInf_ | Inferior Parietal Lobule | DMN,FPN |
| SupraMarg_ | Supramarginal Gyrus | FPN |
| Angular_ | Angular Gyrus | FPN |
| Precuneus_ | Precuneus | DMN,FPN,O |
| ParacentLob_ | Paracentral Lobule | FPN |
| Caudate_ | Caudate Nucleus |  |
| Putamen_ | Putamen |  |
| Pallidum_ | Globus Pallidus |  |
| Thalamus_ | Thalamus | FPN,O |
| Heschl_ | Transverse Temporal Gyrus |  |
| TempSup_ | Superior Temporal Gyrus |  |
| TempPoleSup_ | Superior Temporal Pole |  |
| TempMid_ | Middle Temporal Gyrus |  |
| TempPoleMid | Middle Temporal Pole |  |
| TempInf | Inferior Temporal Gyrus |  |

**References**

Bruña R, Maestú F, Pereda E. Phase Locking Value revisited: teaching new tricks to an old dog. [J Neural Eng.](https://www.ncbi.nlm.nih.gov/pubmed/29952757) 2018 Oct;15(5):056011. doi: 10.1088/1741-2552/aacfe4.

Dimitriadis, S. I., Laskaris, N. A., Bitzidou, M. P., Tarnanas, I., and Tsolaki, M. (2015c). A novel biomarker of amnestic MCI based on dynamic Cross-Frequency Coupling patterns during cognitive brain responses. *Front. Neurosci.* 9:350. doi: 10.3389/fnins.2015.00350

Dimitriadis, S. I. (2018a). Complexity of brain activity and connectivity in functional neuroimaging. *J. Neurosci. Res.* 96, 1741–1757. doi: 10.1002/jnr.24316

Dimitriadis, S. I., Routley, B., Linden, D. E., and Singh, K. D. (2018b). Reliability of static and dynamic network metrics in the resting-state: a MEG-beamformed connectivity analysis. *Front. Neurosci.* 12:506. doi: 10.3389/fnins.2018.00506

J.-P. Lachaux, E. Rodriguez, J. Martinerie, F.J.Varela. Measuring phase synchrony in brain signals.Hum. Brain Mapp., 8 (1999), pp. 194-208

C.J. Stam, G. Nolte, A. Daffertshofer. Phase lag index: assessment of functional connectivity from multi channel EEG and MEG with diminished bias from common sources. Hum. Brain Mapp., 28 (2007), pp. 1178-1193

Haufe, S., Nikulin, V. V., Müller, K.-R., Nolte, G., 2013. A critical assessment of connectivity measures for EEG data: A simulation study. Neuroimage 64, 120–133. https://doi.org/10.1016/J.NEUROIMAGE.2012.09.036

Porz, S., Kiel, M., Lehnertz, K., 2014. Can spurious indications for phase synchronization due to superimposed signals be avoided? Chaos An Interdiscip. J. Nonlinear Sci. 24, 033112. https://doi.org/10.1063/1.4890568

Quiroga, R.Q., Arnhold, J., Grassberger, P., 2000. Learning driver-response relationships from synchronization patterns. Phys. Rev. E 61, 5142–5148. https://doi.org/10.1103/PhysRevE.61.5142

Smitha, K.A., Akhil Raja, K., Arun, K.M., Rajesh, P.G., Thomas, B., Kapilamoorthy, T.R., Kesavadas, C., 2017. Resting state fMRI: A review on methods in resting state connectivity analysis and resting state networks. Neuroradiol. J. 30, 305–317. https://doi.org/10.1177/1971400917697342
